# Evening complex component ELF3 interacts with LUX proteins to repress soybean root nodulation

**DOI:** 10.1111/pbi.70053

**Published:** 2025-03-17

**Authors:** Bohong Su, Hong Li, Ke Zhang, Haiyang Li, Caiyun Fan, Meiling Zhong, Hui Zou, Rujie Li, Liyu Chen, Jing Bo Jin, Mingkun Huang, Baohui Liu, Fanjiang Kong, Zhihui Sun

**Affiliations:** ^1^ Guangdong Key Laboratory of Plant Adaptation and Molecular Design, Innovative Center of Molecular Genetic and Evolution, School of Life Science, Guangzhou University Guangzhou China; ^2^ Key Laboratory of Plant Molecular Physiology, Institute of Botany Chinese Academy of Sciences Beijing China; ^3^ Jiangxi Provincial Key Laboratory of Ex Situ Plant Conservation and Utilization Lushan Botanical Garden, Chinese Academy of Sciences Jiujiang China

**Keywords:** soybean, map‐based cloning, increase nodule number, INN1–LUX, *ENOD40*, negative regulation

## Abstract

Formation of root nodules is a unique hallmark of the symbiotic interaction between legume host plants and rhizobia and is governed by a complex regulatory framework that balances the appropriate orchestration of rhizobial infection and subsequent nodule organogenesis. In contrast to prominent model species such as *Medicago truncatula* and *Lotus japonicus*, research on symbiotic signal transduction in the staple‐crop soybean *Glycine max* remains relatively insufficient. Here, we identified a soybean mutant with ~25% additional root nodules over wild‐type, designated as *increased number of nodules 1* (*inn1*). Through map‐based cloning, *INN1* encodes the EARLY FLOWERING 3a (ELF3a) protein component of the soybean Evening Complex, together with LUX1 and LUX2. *INN1* is co‐expressed with *LUX1* and *LUX2* in roots, and knockout of *INN1* or knockdown of *LUX1* and *LUX2* enhances root nodulation. The function of INN1 in negatively regulating nodulation is genetically and biochemically dependent upon LUXs, as the INN1–LUX complex binds to the promoter of the downstream pro‐nodulation target *ENOD40*, repressing its expression. ELF3a/INN1's repression of root‐nodule formation extends beyond its established roles in diverse above‐ground developmental and physiological processes and offers a theoretical basis for enhancing the biological‐nitrogen fixation capacity of soybean.

## Introduction

Nitrogen (N) is an essential nutrient for plant growth, directly affecting crop development and yield (Tilman, 1999). The nutrient plays a crucial role in photosynthetic efficiency, enhancing crop productivity and maintaining soil quality and health (Poffenbarger *et al*., 2017). The increase in global food production is closely linked to the rise of synthetic nitrogen fertilizer use. However, more than half of the nitrogen applied to farmland is lost to the air and water bodies, leading to severe environmental pollution (Erisman *et al*., 2011; Gu *et al*., 2023; Herridge *et al*., 2008). Therefore, optimizing nitrogen management and its use by crops is key to improving agricultural sustainability and yield.

Symbiotic nitrogen fixation occurs in specialized root nodules of legume roots to accommodate soil‐borne rhizobia attracted by host–plant cues. Nodule formation (nodulation) in legumes is divided into two stages: first, infection by rhizobia and second, organogenesis of the nodule (Nishida and Suzaki, 2018; Yang *et al*., 2022). In the early stages of nodulation, plants release flavonoid or isoflavonoid compounds from their roots. Upon perceiving these cues, rhizobia invade the host plant through gaps in the root epidermal cells or at the position of lateral root formation, and biosynthesize and secrete Nod factors. Nod factors are recognized in the host by receptor protein kinases, including NOD FACTOR RECEPTOR 1 (LjNFR1) and LjNFR5 in *Lotus japonicus*, RECEPTOR‐LIKE KINASE 3 (MtLYK3) and NOD FACTOR PERCEPTION (MtNFP) in *Medicago truncatula*, and NOD FACTOR RECEPTOR (GmNFR1α/β and GmNFR5α/β) in soybean, *Glycine max* (Limpens *et al*., 2003; Madsen *et al*., 2003; Radutoiu *et al*., 2003; Smit *et al*., 2007). After Nod factors bind to the receptors, membrane signalling is activated and transmitted to the cell nucleus via signalling pathways that culminate in activation of pro‐nodulation transcription factors, such as *NODULATION SIGNALLING PATHWAY* (*NSP1/NSP2*) and *NODULE INCEPTION* (*NIN*), thereby initiating the process of nodulation (Liu *et al*., 2019; Schiessl *et al*., 2019; Shrestha *et al*., 2021; Soyano *et al*., 2019).

During the early stages of the interaction between legumes and rhizobia, the expression of *EARLY NODULIN* (*ENOD*) genes plays a key role in the initiation of bacterial infection and/or root‐nodule organogenesis (Nap and Bisseling, 1990). In particular, *ENOD40* is thought to mainly promote the division of cortical cells, thereby inducing the formation of nodule primordia and ensuring proper differentiation and development of the nodule. For example, in *M. truncatula*, overexpression of *ENOD40* increases cortical cell division (Charon *et al*., 1997, 1999). Knockdown of *LjENOD40* inhibits the formation of nodule primordia and subsequent nodule development, but has no apparent effect on the early rhizobial‐infection process (Kumagai *et al*., 2006). Soybean ENOD40 peptides regulate sucrose synthase (SuSy) activity through binding to or guiding the enzyme to specific intracellular locations, thus controlling use of photosynthetic products in the plant (Rohrig *et al*., 2002). Owing to its value as a staple source of protein for food and feed, and for seed oil, soybean nodulation has major effects on seed quality and yield and is of intense interest for agronomic improvement for sustainable agriculture.

EARLY FLOWERING 3 (ELF3) was initially identified as a key regulator of floral induction in *Arabidopsis thaliana*. Mutations in the Arabidopsis *ELF3* gene result in reduced sensitivity to photoperiods, leading to early flowering (Zagotta *et al*., 1996). ELF3 physically interacts with ELF4 and LUX ARRHYTHMO (LUX) to form the Evening Complex, which binds to the promoters of *PHYTOCHROME‐INTERACTING FACTOR 4* (*PIF4*) and *PIF5*, thereby suppressing hypocotyl elongation during the night (Nusinow *et al*., 2011). In maize, ELF3.1 physically interacts with RA2 and ZLF4.2 to form a tri‐protein complex that enhances the binding of RA2 to the *TSH4* promoter, reducing tassel branching (Xie *et al*., 2024). Additionally, ELF3 plays a crucial role in abiotic‐stress‐response pathways, contributing to environmental adaptation (Cheng *et al*., 2020; Sakuraba *et al*., 2017). In soybean, *ELF3* mutations reduce photoperiod sensitivity, enabling adaptation to low‐latitude regions (Lu *et al*., 2017; Yue *et al*., 2017). In summary, ELF3 is a multifunctional regulatory factor involved in diverse physiological processes, highlighting its importance in plant development and environmental adaptation.

Here, we identified a soybean mutant *inn1* exhibiting an increased number of nodules. Map‐based cloning and molecular characterization revealed that soybean *INN1* is homologous to *Arabidopsis thaliana ELF3*. We demonstrate that INN1 function is genetically dependent on LUXs, and the INN1–LUX complex binds to the promoter of the downstream gene *ENOD40* to repress its expression. Our work reveals a regulatory mechanism strongly influencing soybean nodulation. This provides broader insights into the molecular mechanisms underlying establishment of soybean nodulation and offers a theoretical basis for enhancing soybean biological‐nitrogen fixation capacity.

## Results

### The soybean *inn1* mutant has extra root nodules

**Figure 1:**
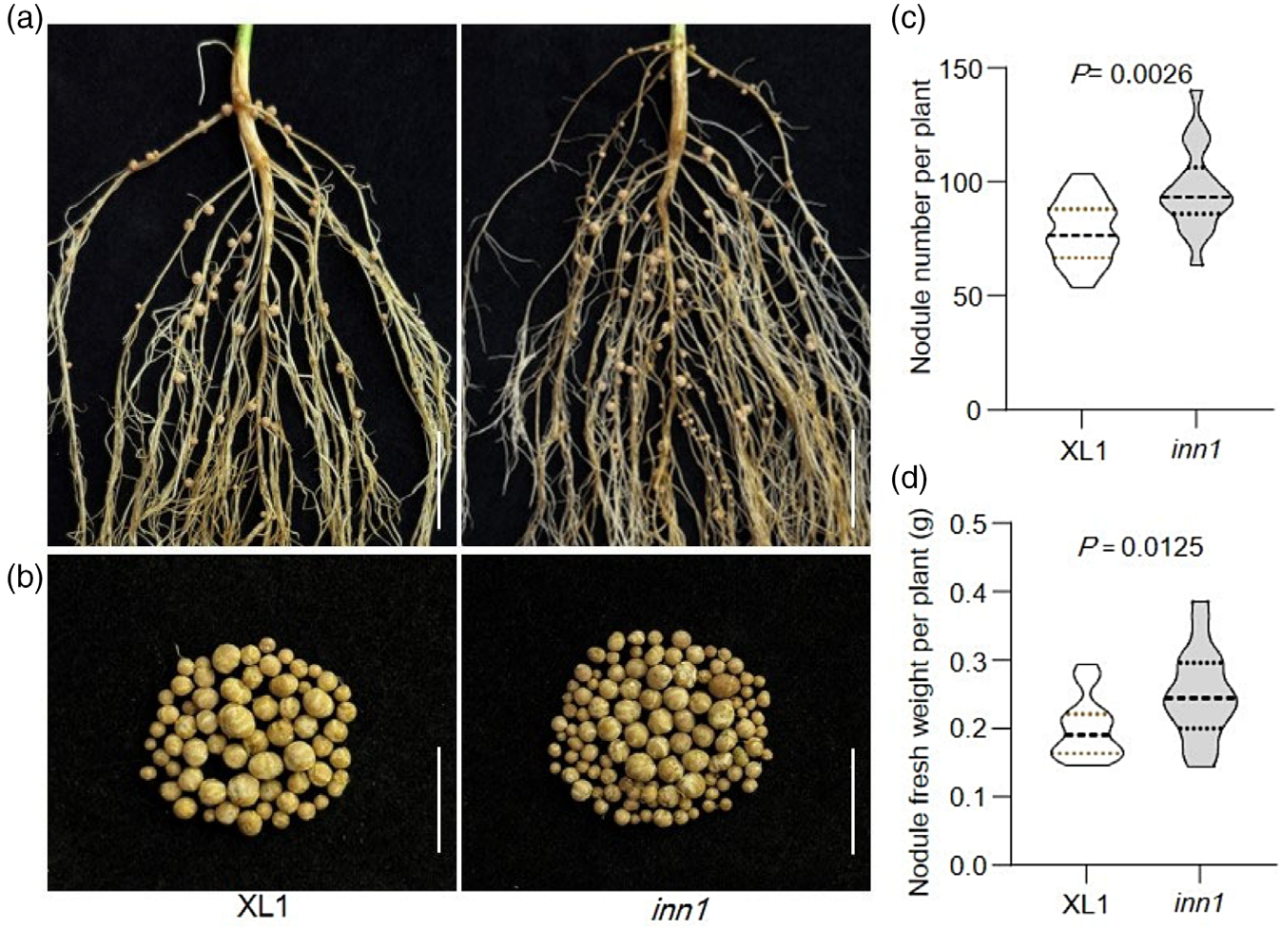
The soybean *inn1* mutant produces more root nodules. (a) Root and nodule phenotypes of wild‐type XL1 and *inn1* at 21 days after inoculation (dai) with USDA110 rhizobium. Bar = 2 cm. (b) Nodule characteristics of wild‐type XL1 and *inn1* at 21 dai. Bar = 4 cm. (c) Nodule number per plant for XL1 and *inn1* (*n* = 18). (d) Nodule fresh weight per plant for XL1 and *inn1* (*n* = 18). For (c, d), data are means ± SD. *P* < 0.05, two‐tailed Student's *t*‐test.

Maintaining the appropriate number of root nodules is a major factor for improving soybean yield and quality (Zhong *et al*., 2024). Excessive nodulation directly leads to soybean growth at the expense of root development and vegetative biomass (Carroll *et al*., 1985). To identify mutants with altered nodule numbers, we screened a gamma‐ray‐induced mutant in the wild‐type cultivar XL1 (Jilinxiaoli1). A mutant with statistically significantly increase in nodule numbers was identified and named *increased nodule number 1* (*inn1*) (Figure 1a,b). Compared to the wild‐type XL1, the *inn1* mutant displays 25% more nodules, accompanied by a higher total‐nodule fresh weight per plant (Figure 1c,d).

To identify the candidate gene underlying the *inn1* mutant responsible for nodule number, we crossed the wild‐type c.v. XL1 and *inn1* mutants to develop an F_2_ segregating population containing 180 individuals (Figure S1). Based on the nodule phenotyping of the progeny, 20 individuals with a high number of nodules and 20 individuals with a low number of nodules were selected, and two extreme‐phenotype pools were constructed. The parents and the two extreme phenotypic pools were subjected to whole‐genome resequencing. After data filtration and screening, 118 016 high‐quality SNPs and 24 015 high‐quality indels were obtained for subsequent bulked‐segregant analysis by sequencing (BSA‐seq) and analysis (Tables S3 and S4). SNP sites with statistically significant genotype differences between the two extreme‐phenotype pools were selected. The Euclidean Distance (ED) value for each site was calculated. To minimize background noise, the original ED values were raised to the fifth power to enhance the signal (Hill *et al*., 2013). Ultimately, a large candidate region was identified on chromosome 4, spanning the physical position from 2.14 to 45.07 Mb (Figure S2).

To narrow down the candidate region, Wm82 was crossed with the *inn1* mutant to obtain an F_2_ segregating population consisting of 241 individuals (Figure S3). Simple‐sequence‐repeat (SSR) molecular markers within the candidate region were selected for polymorphism screening, and six markers showing polymorphisms between the parents were identified. These markers were then used to genotype the F_2_ progeny, and QTL mapping was conducted in conjunction with the nodule number phenotype. This analysis narrowed the candidate region to a 3.20–4.43 Mb interval (Figure S4a,b).

Next, we compared resequencing data from XL1 and *inn1* to identify differential SNPs and indels within the candidate region, toward identifying potential target genes. Within the narrowed 1.23‐Mb physical region, three indels were identified between XL1 and *inn1* (Table S2). We then investigated whether these indels had any functional impact on annotated genes. A 20‐bp deletion was found in exon four of *Glyma.04G050200*, predicted to result in premature translation termination of the protein product (668‐amino acid), compare with 715 amino acid in wild‐type (Figure S4c,d). The other two indels were located in intergenic regions and did not affect protein coding (Table S2). Therefore, *Glyma.04G050200* was designated as the candidate gene underlying *INN1*.

### *INN1* encodes an ELF3 homologue and is a negative regulator of soybean nodulation

*Glyma.04G050200* encodes the hydroxyproline‐rich glycoprotein EARLY FLOWERING 3a (ELF3a), a component of the Evening Complex with established roles in latitudinal adaptation and juvenility in legumes (Bu *et al*., 2021; Rubenach *et al*., 2017; Yue *et al*., 2017) and as a thermosensor in Arabidopsis (Jung *et al*., 2020).

**Figure 2:**
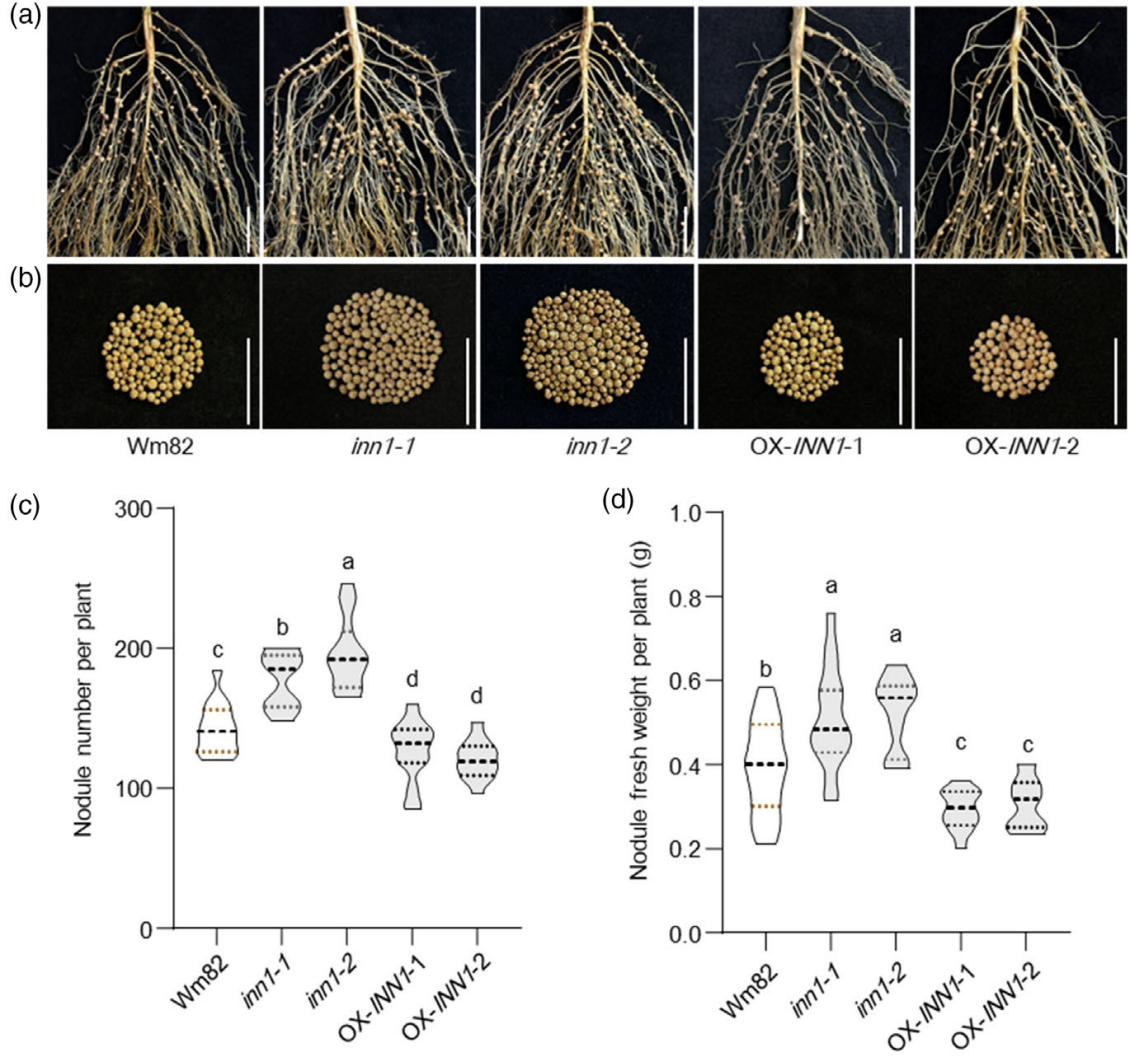
INN1 negatively regulates soybean nodule formation. (a, b) Root (a) and nodule (b) characteristics of wild‐type c.v. Wm82, *inn1‐1*, *inn1‐2* and two independent transgenic *INN1*–*6xHA* overexpression lines. Scale bar = 2 cm. (c) Nodule number per plant (*n* = 18). (d) Nodule fresh weight per plant (*n* = 18). Under low‐nitrogen conditions, nodule number and weight were measured for each plant at 21 dai. All data are presented as means ± SD. Different letters represent statistically significant differences between means determined by one‐way ANOVA (*P* < 0.05).

To verify the function of *INN1* in the context of soybean nodulation, knockout mutants obtained by CRISPR–Cas9 in previous studies were investigated (herein named *inn1‐1* and *inn1‐2*) (Li *et al*., 2024; Qin *et al*., 2023). The *inn1‐1* mutant contains two deletions from exon 4, leading to a premature stop codon and encoding a 560 amino acid protein (versus 715 amino acid in wild‐type). The *inn1‐2* mutant has a 2‐bp deletion from exon 2, resulting in a premature stop codon and encoding a 153 amino acid protein (Figure S5a–d). Compared to the wild‐type Wm82, both knockout mutants had a statistically significant increase in nodule number and a substantial nodule weight (Figure 2), similar to that of the gamma‐ray‐induced *inn1* mutant (Figure 1c,d).

To further characterize the function of *INN1* in nodulation, we investigated phenotypes of two independent transgenic lines overexpressing *INN1* fused to the 6*x*HA tag (hereinafter ‘*INN1–6xHA*’) driven by the CaMV *35S* promoter in the c.v. Wm82 background (Zhao *et al*., 2024). Both transgenic overexpression lines overexpress *INN1* at the transcript level (Figure S5e) and INN1–6×HA accumulation at the protein level is detectable by western blot with anti‐HA antibodies (Figure S5f). *INN1–6xHA* plants had statistically significantly fewer nodules per root compared to wild‐type c.v. Wm82, along with a substantial reduction in nodule weight (Figure 2). These results indicate that *INN1* functions as a negative regulator of soybean nodulation.

### *INN1* and *LUXs* exhibit overlapping spatial expression patterns during the early stages of nodulation

INN1/ELF3a is a component of the Evening Complex, which plays a key role in regulating the circadian clock and photoperiod responses (Lu *et al*., 2017). In soybean, ELF3a, along with LUX1 and LUX2, forms the Evening Complex to regulate photoperiod sensitivity and adaptation (Bu *et al*., 2021; Rubenach *et al*., 2017; Yue *et al*., 2017). To investigate whether these complex‐member genes overlap in terms of spatial and temporal expression patterns and potentially contribute to root nodulation, their expression was analysed in various tissues. All three genes were detected by RT–qPCR in roots, stems, leaves, flowers, seed pods and nodules of inoculated plants, suggesting their involvement in multiple physiological processes (Figure S6a–c).

GUS reporter constructs were developed by fusing the *INN1*, *LUX1* and *LUX2* promoters to *GUS* and used for transient transformation of hairy roots. Histochemical staining revealed that *INN1*, *LUX1* and *LUX2* promoter activities were consistent. During the early stages of nodulation, GUS expression from *INN1*, *LUX1* and *LUX2* promoters was mainly localized in the stele and nodule primordium, indicating potential roles in nodule formation and development (Figure S6d–f). These findings suggest that *INN1*, *LUX1* and *LUX2* may regulate nodule development and contribute to the control of nodule number.

### The INN1–LUX complex coordinately regulates soybean nodulation

**Figure 3:**
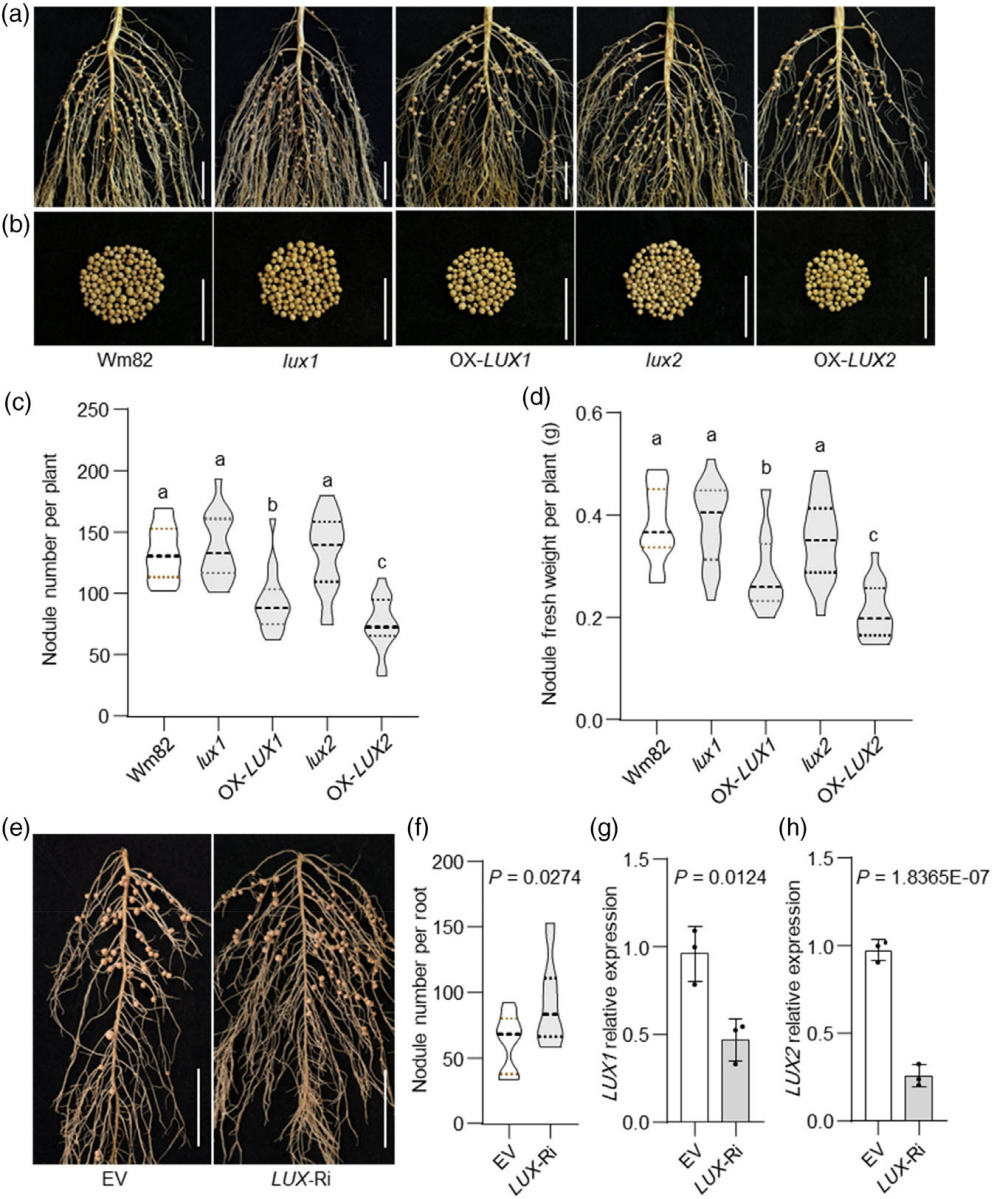
LUX negatively regulates soybean nodulation. (a, b) Root (a) and nodule (b) characteristics of wild‐type c.v. Wm82, *lux1*, *lux2* and transgenic *LUX1–FLAG* and *LUX2–FLAG* overexpression lines. Scale bar = 2 cm. (c) Nodule number per plant (*n* = 18). (d) Nodule weight per plant (*n* = 18). (e) Nodule phenotypes of transgenic hairy roots transformed with the empty‐vector control and a *LUX* RNAi vector at 28 dai. Scale bar = 2 cm. (f) Nodule number per hairy root (*n* = 15). (g, h) RT–qPCR of *LUX1* (g) and *LUX2* (h) expression levels in hairy roots carrying the empty vector (EV) and *LUX* RNAi vector (*n* = 3 biological replicates ± S.D.). Relative expression was normalized to *ACTIN11*. Under low‐nitrogen conditions, nodule number and weight were measured for each plant at 21 dai. Data are means ± SD, with individual values shown as dots in g and h. For c, and d, one‐way ANOVA was used to determine the of effects of genotype on nodule traits (*P* < 0.05) and different letters indicate statistically significant differences. For f–h, the effect of genotype on means was determined by two‐tailed Student's *t*‐tests (*P* < 0.05).

Given the overlap in gene‐expression patterns for *INN1* with *LUX1*/*2* and their known involvement in the Evening Complex, we therefore sought to further investigate the contribution of LUX1/2 to nodule number by exploiting knockout mutants and transgenic *LUX–FLAG* overexpression lines described previously (Bu *et al*., 2021). Under low‐nitrogen conditions, *lux1* and *lux2* single mutants did not show obvious differences in nodule phenotypes compared to Wm82 controls, suggesting these genes do not individually have drastic effects on nodulation (Figure 3a–d). The *lux1 lux2* double mutant generated by CRISPR exhibits an excessively extended growth period and flower abortion and very few seeds (Bu *et al*., 2021). Thus, we used RNA interference (RNAi) to knockdown of both *LUX1* and *LUX2* expression in hairy roots. The results showed a statistically significant increase in nodule number in knockdown lines (Figure 3e–h). Furthermore, both *LUX1–FLAG* and *LUX2–FLAG* overexpression lines exhibited statistically significantly fewer nodules compared to Wm82, indicating *LUX1* and *LUX2* inhibit soybean nodulation (Figure 3a–d). These results suggest that *LUX1* and *LUX2* act as negative regulators of soybean nodulation.

**Figure 4:**
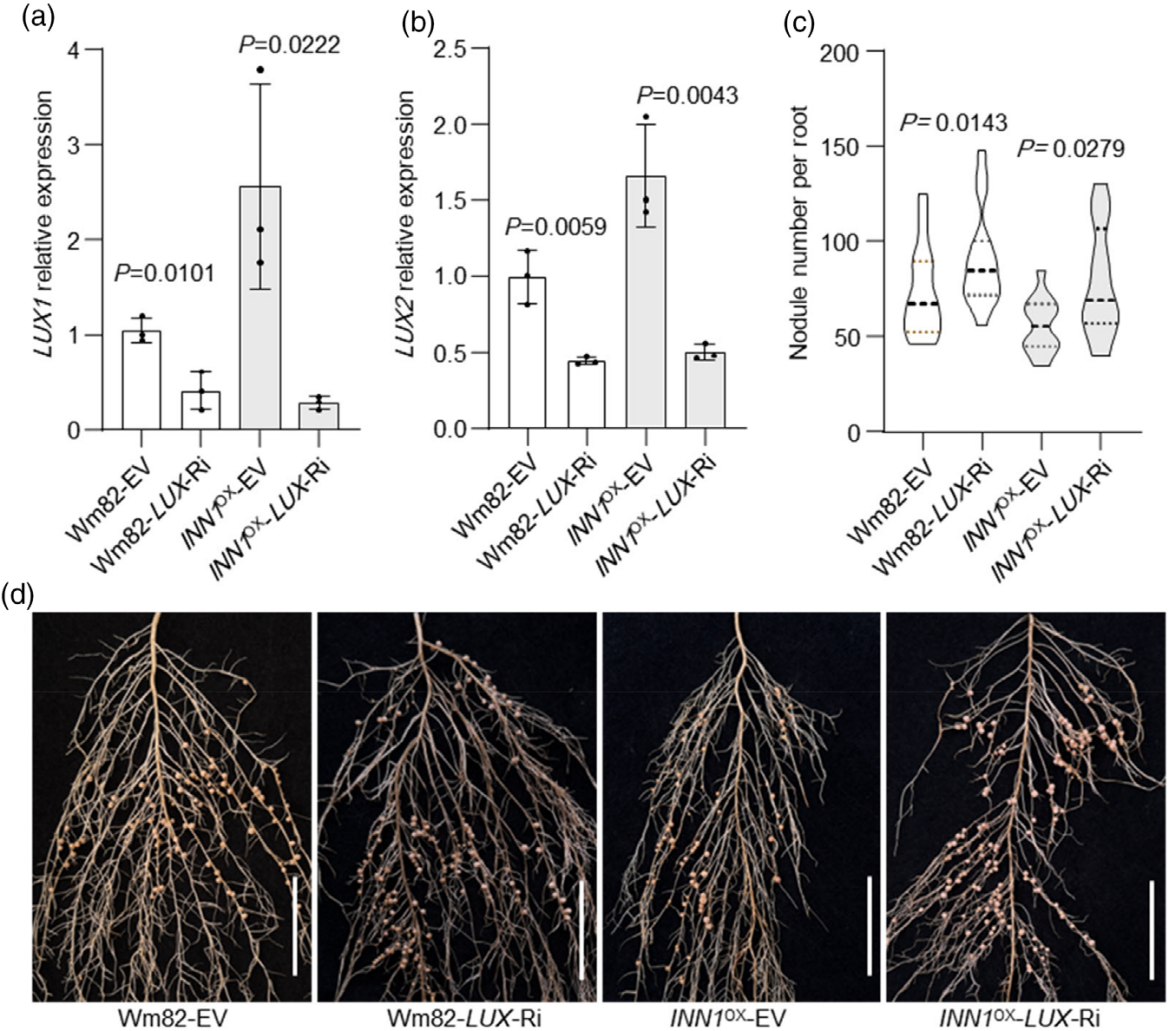
INN1 functions in a LUX‐dependent manner. (a, b) RT–qPCR of *LUX1* and *LUX2* expression levels in transgenic hairy roots carrying the empty vector (EV) and *LUX* RNAi vector (*n* = 3 biological replicates ± S.E. with individual plant values shown as dots). Relative expression was normalized to *ACTIN11*. (c) Root‐nodule number per hairy root (*n* = 12). (d) Root‐nodule phenotypes of transgenic hairy roots expressing the empty vector (EV) and *LUX* RNAi vector in Wm82 and OX‐INN1–1 backgrounds under low‐nitrogen conditions at 28 dai. Scale bar = 2 cm. The effect of genotype on nodule number or relative expression was determined by one‐way ANOVA (*P* < 0.05).

To clarify the genetic relationship between *INN1* and *LUXs*, RNAi was used to simultaneously knock down the expression of *LUX1* and *LUX2* in hairy roots of *INN1* overexpression lines (Figure 4a,b). This down‐regulation was sufficient to significant increase nodule number compared to the empty‐vector control group (Figure 4c,d). This result suggests that INN1 may depend on LUXs to exert its regulatory function.

### *ENOD40* is a downstream target of the INN1–LUX complex for nodulation

**Figure 5:**
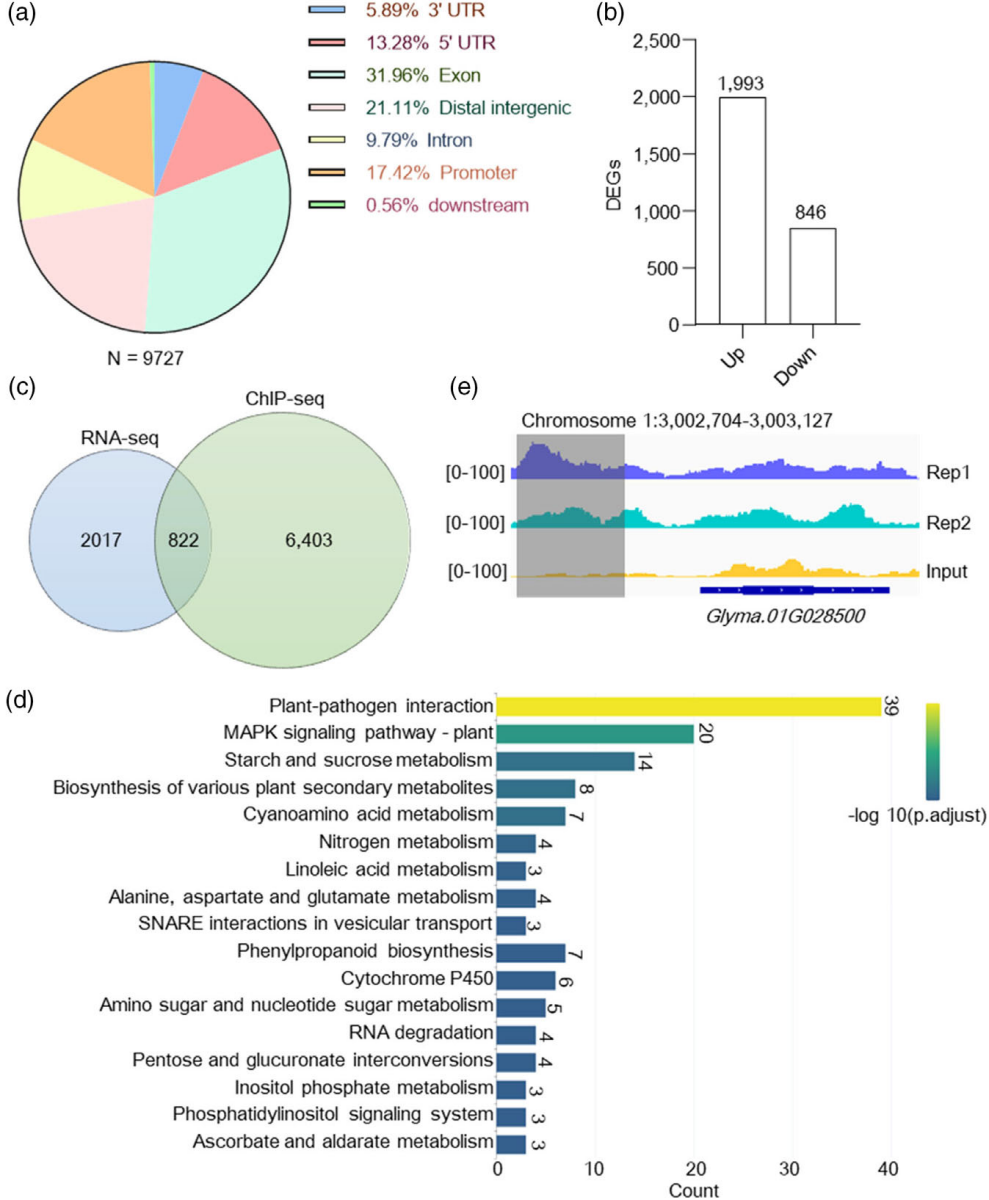
Identification of INN1–LUX‐regulated genes through ChIP‐seq and RNA‐seq analysis. (a) Genomic distribution of ChIP‐seq binding peaks from data for INN1–LUX. (b) Number of differentially expressed genes (DEGs) identified in RNA‐seq analysis of Wm82 and *inn1‐2* transcriptomes. (c) Integration of RNA‐seq and ChIP‐seq data reveals the number of candidate direct targets of the INN1–LUX complex. (d) KEGG pathway analysis of genes overlapping between ChIP‐seq and RNA‐seq data, only the top 17 KEGG pathways, ranked by p.adjust value, are shown in the bar chart, while the remaining pathways are listed in Table S7. (e) ChIP‐seq reads for LUX–FLAG occupancy in a region of chromosome 1 surrounding the *ENOD40* (*Glyma.01G028500*) locus. Two biological replicates and the input‐only control are plotted. The grey box depicts binding region, blue and cyan peaks indicate *ENOD40* genome browser tracks in the replicates, and yellow peaks indicate *ENOD40* gene genome browser tracks in the input control. Normalized read counts are represented in brackets.

LUX proteins contain a SHAQYF‐type GARP transcription‐factor MYB domain, which mediates interactions between the Evening Complex and target‐gene promoters by binding to the LUX binding site (LBS, GATWCK or GATWYG) or consensus LBS (GATWCG) (whereby W represents A or T, K represents G or T, Y represents C or T) (Helfer *et al*., 2011; Nusinow *et al*., 2011). To identify downstream genes regulated by the INN1–LUX complex in soybean, we used a 3‐d‐old Wm82 and OX‐LUX2 transgenic line inoculated seedlings root for chromatin immunoprecipitation followed by sequencing (ChIP‐seq) (Figure S7, Tables S5–S7). A total of 9727 binding peaks were identified, spanning 7225 annotated genes, with 17.42% of the peaks located in promoter regions (Figure 5a). Next, RNA‐seq analysis was performed on 3‐d‐old Wm82 and *inn1‐2* inoculated seedlings root, from which 2839 differentially expressed genes (DEGs) were identified (*P*‐value <0.05, FC ≥2). Of these, 1993 genes were up‐regulated and 846 were down‐regulated (Figure 5b).

By integrating ChIP‐seq and RNA‐seq data, 822 genes were identified as potential targets regulated by the INN1–LUX complex (Figure 5c). Of the 822 genes, 741 showed up‐regulation, and 81 showed down‐regulation. KEGG pathway enrichment analysis indicated that these DEGs are mainly involved in regulating plant–microbe interactions. Genes associated with starch and sucrose metabolism, specialized metabolite biosynthesis and nitrogen metabolism were also highly enriched in KEGG pathways (Figure 5d, Table S8), all of which are implicated in establishment and maintenance of symbiotic nitrogen fixation in legume root nodules.

RNA‐seq data revealed statistically significant up‐regulation of symbiotic‐signalling marker genes in the *inn1‐2* mutant, including *ENOD40*, *NIN1a*, *NIN2a* and *NIN2b* (Table S9). ChIP‐seq indicated LUX*–*FLAG binding peaks were exclusively found in the promoter region of *ENOD40* (chromosome 1: position 3 002 704–3 003 127) (Figure 5e, Table S10). These findings suggest that the INN1–LUX complex regulates symbiotic signalling by binding to the *ENOD40* promoter.

### The INN1–LUX complex binds to and represses the expression of *ENOD40*

**Figure 6:**
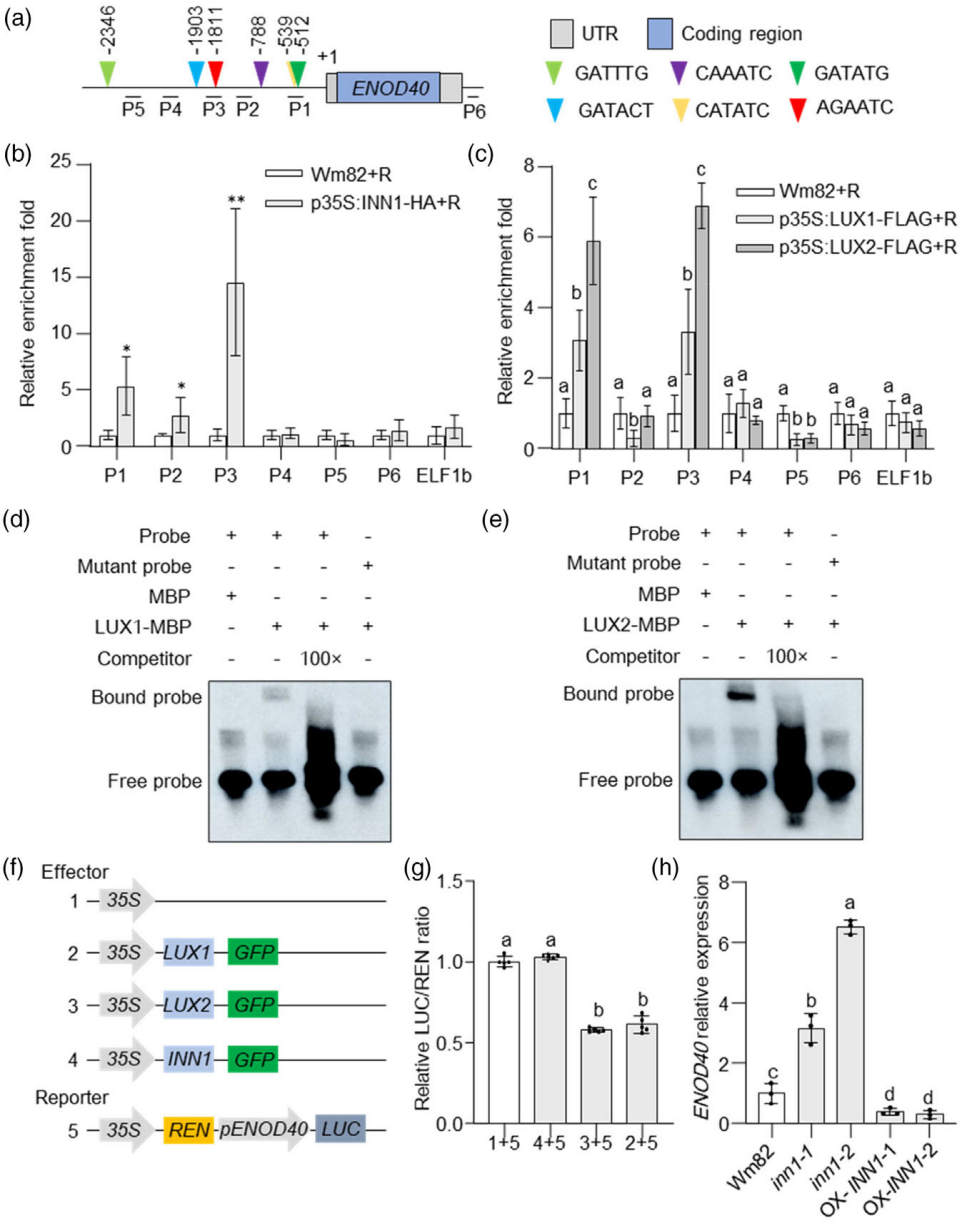
The INN1–LUX complex directly binds to the *ENOD40* promoter to repress its transcription. (a) Schematic of the *ENOD40* promoter showing the regions tested for enrichment by ChIP–qPCR (P1–P6) and EMSA. Coloured triangles indicate the *cis*‐regulatory LUX binding sites. (b) ChIP–qPCR of INN1–HA binding to the *ENOD40* promoter in stable‐transgenic lines following rhizobial inoculation. Asterisks indicate statistical significance compared to the respective negative control group (Wm82) (Student's *t*‐test: *P < 0.05; **P < 0.01). (c) LUX1–FLAG and LUX2–FLAG binding to the *ENOD40* promoter in stable‐transgenic lines following rhizobial inoculation. The *ELF1b* promoter was used as a negative control. (d, e) EMSA of LUX1–MBP (d) and LUX2–MBP (e) binding to the LUX binding sites (P1) present in the *ENOD40* promoter. MBP alone is the control protein. (f) Schematic of the *INN1*, *LUX1* and *LUX2* effector and *ENOD40* dual‐reporter vectors used for transient‐expression assays in *N. benthamiana* leaves. (g) LUC/REN activity for the *ENOD40* dual‐reporter construct effected by control, LUX1 and LUX2 to repress *ENOD40* promoter activity. Data are presented as means ± SD of *n* = 5 biological replicates and normalized to LUC/REN for the empty‐vector control effector. Different letters represent statistically significant differences determined by Kruskal–Wallis test, *P* < 0.05. (h) *ENOD40* expression in 3‐days old root isolated from wild‐type c.v. Wm82, *inn1‐1*, *inn1‐2* and two transgenic *INN1‐6xHA* lines. Different letters represent statistically significant differences determined by one‐way ANOVA, *P* < 0.05.

To confirm whether the INN1–LUX complex directly binds to the *ENOD40* promoter, the presence of LUX binding sites within the 2‐kb promoter region of *ENOD40* was investigated. Six potential binding sites were identified, designated P1–P6 (Figure 6a). Chromatin immunoprecipitation coupled to qPCR (ChIP–qPCR) showed that INN1–HA binds to the P1, P2 and P3 regions of the *ENOD40* promoter, which comprise LUX binding sites (Figure 6b). Similarly, both OX‐LUX1 and OX‐LUX2 were found to bind to the P1 and P3 regions of the *ENOD40* promoter (Figure 6c). These results confirm that the INN1–LUX complex can bind to the *ENOD40* promoter in planta. To further investigate whether LUX1, LUX2 or INN1 directly bind to the *ENOD40* promoter, an electrophoretic mobility shift assay (EMSA) was performed. The results showed no shifted signal in gel supported that INN1–MBP does not directly bind *ENOD40* promoter (Figure S8). Recombinant LUX1–MBP and LUX2–MBP specifically bind to a labelled probe containing LUX binding sites (as in the *ENOD40* promoter), supporting the hypothesis that LUX1/2 directly bind to the *ENOD40* promoter to regulate its expression (Figure 6d,e).

To examine the putative negative regulatory role of the INN1–LUX complex in *ENOD40* transcription, transient *trans*‐activation reporter assays were carried out using INN1, LUX1 and LUX2 candidate effectors (all fused to GFP) co‐transformed with the *ENOD40* promoter fused to the *LUC* reporter gene (Figure 6f). Co‐transformation of INN1 with *ENOD40*_*pro*_ *:LUC* showed no statistically significant change in LUC/REN activity compared to the empty‐effector control. However, co‐transformation of LUX1 or LUX2 with *ENOD40*_*pro*_ *:LUC* resulted in a statistically significant reduction in LUC/REN activity, suggesting that INN1 inhibits *ENOD40* promoter activity through LUX1 and LUX2 (Figure 6g).

To confirm whether the INN1–LUX complex regulates *ENOD40* transcription, *ENOD40* expression was assayed following rhizobial inoculation in different soybean genotypes. Loss of *INN1* statistically significantly increased *ENOD40* expression compared to the wild‐type, while *INN1* overexpression statistically significantly reduced it (Figure 6h). Moreover, in *LUX* RNAi hairy root lines, *ENOD40* expression was up‐regulated, whereas *LUX1*/*2* overexpression led to a *ENOD40* down‐regulation (Figures S9 and S10). These findings suggest that the INN1–LUX complex binds to the *ENOD40* promoter and regulates its expression by repression.

**Figure 7:**
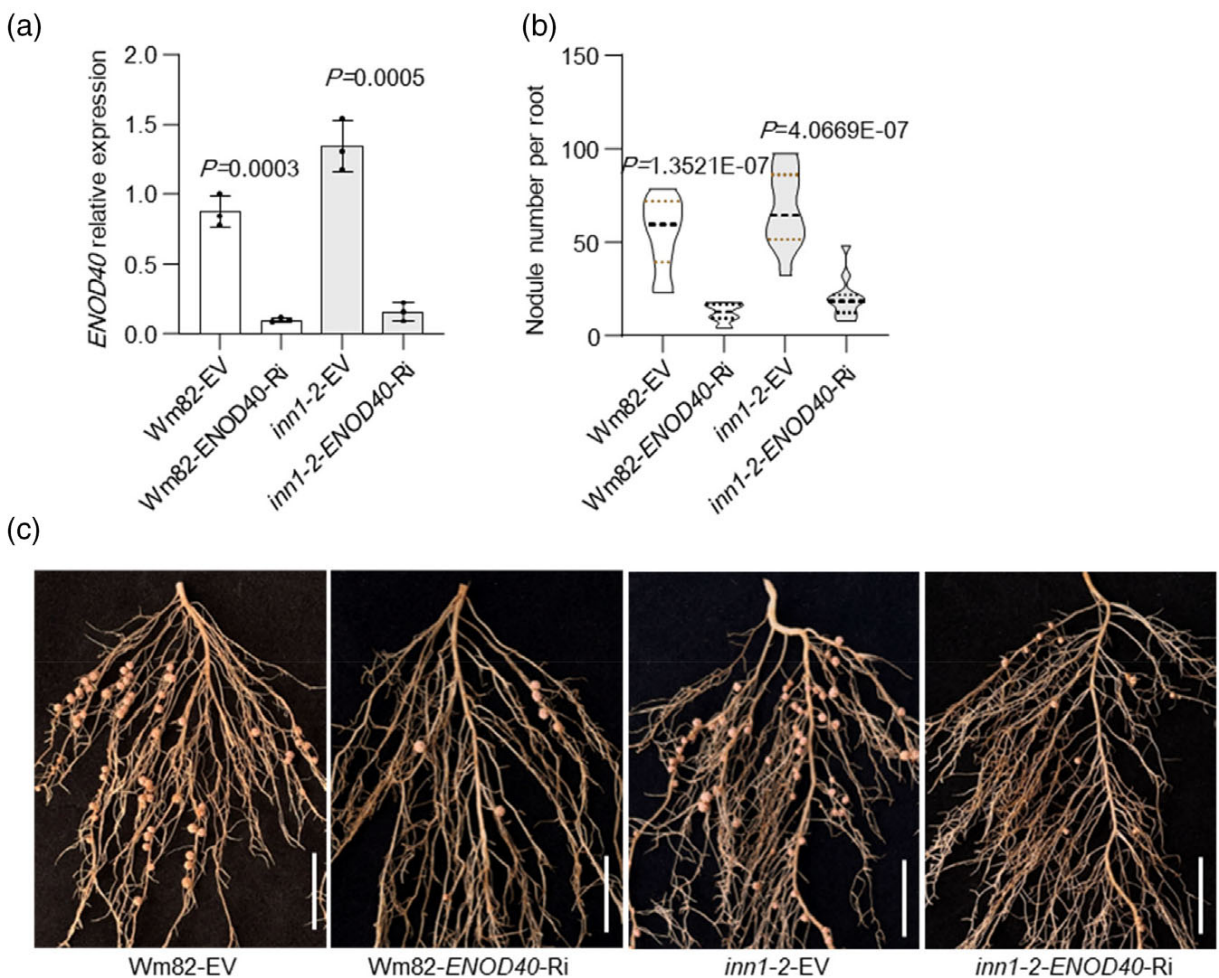
ENOD40 functions downstream of *INN1* in nodulation. (a) RT–qPCR of *ENOD40* expression levels in transgenic hairy roots carrying the empty‐vector (EV) control and *ENOD40* RNAi vector in wild‐type c.v. Wm82 and *inn1‐2* backgrounds (*n* = 3 biological replicates). *ACTIN11* was used to normalize relative expression. (b) Nodule number per hairy root for genotypes as in (C) (*n* = 12). For a and b, data are means ± SD with individual plant values shown as dots. *P* < 0.05, two‐tailed Student's *t*‐test. (c) Nodule phenotypes of transgenic hairy roots expressing empty‐vector (EV) control and *ENOD40* RNAi vectors in wild‐type c.v. Wm82 and *inn1‐2* backgrounds at 28 dai. Scale bar = 2 cm.

To further explore the genetic relationship between *INN1* and *ENOD40*, RNAi was used to down‐regulate *ENOD40* expression in hairy roots in both Wm82 and *inn1‐2* backgrounds. Following *ENOD40* down‐regulation expression (Figure 7a), nodule number for lines in both Wm82 and *inn1‐2* backgrounds was statistically significantly lower compared to empty‐vector control roots (Figure 7b,c). This indicates that ENOD40 functions as a downstream effector in INN1‐mediated regulation of nodule formation.

## Discussion

The symbiotic relationship between legumes and rhizobia, which facilitates the conversion of atmospheric nitrogen into ammonium provides a crucial nitrogen source for plants, supporting their growth and development (Herridge *et al*., 2008; Oldroyd, 2013). This nitrogen‐fixing symbiosis is particularly significant in soybeans, from which approximately 70% of the plant's nitrogen is sourced from this relationship with rhizobia. This not only ensures adequate nitrogen nutrition for the host plant but also plays a vital role in soybean‐seed yield and quality. Increasing the number of nodules in soybeans enhances biological‐nitrogen fixation, improves nitrogen use efficiency, promotes photosynthesis, maintains the carbon‐nitrogen balance and ultimately boosts soybean yield and quality (Zhong *et al*., 2024). However, an excess of extra nodules results in major growth defects (Carroll *et al*., 1985). In this study, *INN1* was identified in a screen in which the corresponding mutant has more nodules, highlighting its critical role in regulating nodule formation. Functional characterization revealed *INN1* acts in a gene‐family‐dependent manner, specifically relying on *LUX1* and *LUX2* family members, to regulate the expression of *ENOD40*, thereby participating in nodule development and regulation of nodule quantity. Based on these findings, we propose a new genetic‐regulatory module INN1–LUX–ENOD40 as a mechanism contributing to nodule initiation in soybean. Notably, a role for the INN1*–*LUX complex in root nodulation has not been reported to our knowledge in other legumes. This not only provides insights into the molecular mechanisms controlling nodule number in soybean, but also offers valuable clues for further research on the biological‐nitrogen fixation capacity of other legumes.

In soybean, *INN1* encodes ELF3a, which is involved in the floral transition, shade avoidance and water transport (Andrade *et al*., 2022; Bu *et al*., 2021; Nusinow *et al*., 2011). ELF3 also plays a critical role in regulating plant growth and development, as well as enhancing abiotic‐stress tolerance (Cheng *et al*., 2020; Dong *et al*., 2021a). Arabidopsis ELF3 functions as a thermosensor, signalling environmental‐temperature changes through its prion‐like domain (PrD), thus positioning ELF3 as a heat sensor (Jung *et al*., 2020). Here, we demonstrated that INN1 acts as a negative regulator of nodulation, with more nodules observed in both the *inn1* gamma‐induced mutant and those obtained by gene editing (Figure 2). In soybean, *elf3a* mutants have extended juvenile stages and enhanced yield under short‐day conditions that promote flowering (Li *et al*., 2024; Lu *et al*., 2017). Similarly, *inn1* mutants also exhibited greater biomass at maturity than XL1 (Figure S11). The number of nodules formed and the amount of nitrogen fixed in a nodulated soybean plant increase during vegetative growth, reaching a peak at the flowering stage before seed development begins. After flowering, nodules continue to supply nitrogen, supporting both reproductive and vegetative growth, until they senesce (Nleya *et al*., 2013; Yun *et al*., 2023). It is plausible that the *INN1* knockout mutant, which leads to a long juvenile period and larger vegetative biomass at maturity, also results in higher nitrogen demand, though this remains to be confirmed experimentally (Li *et al*., 2024). Consequently, the extra nodules can assist toward meeting the nitrogen demands of juvenile soybean plants. In Arabidopsis, the length of the polyglutamine (polyQ) repeat sequence at the C‐terminus of ELF3 correlates with heat responsiveness (Jung *et al*., 2020). An open question remains whether the extent of C‐terminal truncations of INN1 will impact nodule numbers, potentially revealing an unknown regulatory mechanism (Figure S5c). In *M. truncatula*, *LUX* regulates nodule numbers, likely through the indirect modulation of MtLHY. Both *M. truncatula lux* and *lhy* mutants exhibit have fewer nodules and altered expression of genes involved in amino acid and nitrogen metabolism (Kong *et al*., 2022). In contrast, in soybean, *LUX1*/*2* function as negative regulators of nodulation, with down‐regulation of *LUX1* and *LUX2* leading to extra nodules (Figure 3e–h). However, the loss of a single *LUX* gene does not impact soybean nodule number, likely due to functional redundancy among family members, a phenomenon also observed for *LUXs* in regulating flowering time (Bu *et al*., 2021). An overlapping spatiotemporal expression pattern of *INN1* and *LUXs* was observed in soybean roots and nodules, providing support for the hypothesis that they jointly regulate nodule number (Figure S6d–f). Genetic validation further confirmed that INN1 regulates nodule number in a LUX‐dependent manner (Figure 4).

The symbiotic relationship between rhizobia and legume hosts relies on the activation of rhizobia‐induced signalling pathways and the precise regulation of key transcription factors during the formation of the nodule primordium (Dong *et al*., 2021b; Downie, 2014; Endre *et al*., 2002; Marsh *et al*., 2007; Peck *et al*., 2006). This study extends the breadth of soybean nodulation‐initiation networks and revealed the critical role of the INN1–LUX complex in this, but the specific downstream target genes that mediate its negative regulation of nodulation remain to be identified and characterized. To address this, RNA‐seq and ChIP‐seq combined to screen for candidate downstream targets, from which *ENOD40* was identified (Figure 5). *ENOD40*, an early marker gene induced by rhizobia, plays an essential role in nodulation. Its expression in soybean is regulated by several factors, including *NFYA‐C*, *NNC1* and *SPL9d* (Ma *et al*., 2019; Xu *et al*., 2021; Yun *et al*., 2022; Zhang *et al*., 2022). *ENOD40* is rapidly induced upon inoculation with rhizobia or purified nodulation factors, wherein *ENOD40* functions as an intercellular signalling molecule or participates in regulating carbon metabolism during nodulation (Campalans *et al*., 2004; Yang *et al*., 1993). In this study, multiple LUX binding sites were identified in the *ENOD40* promoter, providing the necessary conditions for the INN1–LUX complex to bind (Figure 6a–e). Similarly, during flowering, the ELF3a and LUX complexes typically regulate the transcription of downstream genes by binding to LUX binding sites (Fang *et al*., 2021). Using hairy roots expression experiments, we validated that down‐regulation of *ENOD40* also results in fewer nodules (Figure 7). Consistent with our results, previous work has shown that reducing *ENOD40* expression in *L. japonicus* suppresses nodulation (Kumagai *et al*., 2006). Additionally, in the soybean *inn1‐2* mutant, attenuated *ENOD40* leads to a reduction in nodule number, further supporting the genetic interaction between *INN1* and *ENOD40* and their important role in soybean nodulation.

Here, we have demonstrated that the INN1–LUX module acts as a negative regulator of *ENOD40*. However, it remains unclear whether the inhibitory effect of INN1–LUX on the *ENOD40* promoter is sustained over time or occurs as a ‘brake’ after rhizobia‐induced activation. Based on these data, we hypothesize that the INN1–LUX–ENOD40  module likely acts after the rhizobia‐induced activation of symbiotic marker genes, as *ENOD40* is typically not expressed before rhizobial induction. While ChIP–qPCR was used to confirm that INN1–LUX does not detectably bind to the *ENOD40* promoter in uninoculated plants rhizobia (Figure S12), more experimental evidence is needed to further support this conclusion.

### Plant materials and growth conditions

Soybean (*Glycine max* L.) cultivars Williams 82 (Wm82) and Jilinxiaoli1 (XL1) were selected as wild‐type materials. The *INN1* CRISPR mutants and overexpression lines were generated in the c.v. Wm82 background (Li *et al*., 2024; Qin *et al*., 2023). *LUX1* and *LUX2* CRISPR mutants and overexpression lines were described previously and are in the Wm82 background (Bu *et al*., 2021).

The *inn1* mutant was obtained by γ‐ray mutagenesis of wild‐type c.v. XL1. F_2_ genetic‐segregation populations were constructed for QTL mapping using Wm82 as the male parent and *inn1* as the female parent and for BSA‐seq analysis using XL1 as the male parent and *inn1* as the female parent.

Soybean and *Nicotiana benthamiana* seedlings were grown in a growth chamber under a 28 °C, 16‐h light/8‐h dark photoperiod with humidity maintained at 70%.

### BSA‐seq and QTL mapping

Genomic DNA was extracted from the leaves of F_2_ individuals from the XL1 × *inn1* population using the NuClean Plant Genomic DNA (ComWin Biotech Co., Ltd). After scoring nodule number in all F_2_ individuals, 20 extreme individuals each with a high and low nodule count were selected. Equal amounts of DNA were extracted from these individuals and pooled to construct DNA pools, which were then used for subsequent BSA‐seq analysis. Similarly, in the Wm82 × *inn1* population, DNA was extracted from all F_2_ individuals after nodule evaluation. For BSA‐seq analysis, a threshold for association analysis was set using fitted values of all loci plus three times the standard deviation (median + 3 SD), resulting in a threshold of 0.01.

Based on the BSA‐seq analysis results, SSR markers with high polymorphisms on chromosome 4 of parent were selected for genotyping the progeny of the population. QTL analysis was performed using IciMapping software (https://isbreedingen.caas.cn/software/qtllcimapping/294607.htm) to further narrow down the candidate region (Meng *et al*., 2015). A LOD value of 2.5 was considered the minimum threshold for statistically significant QTL identification. The marker primer used for genotyping are listed in Table S1.

### Nodule–phenotype evaluation

The seeds used for nodule phenotyping in both population and genetically modified plants were sterilized with chlorine gas for 8 h and subsequently germinated in sterile vermiculite. Healthy, plump seeds were sterilized with chlorine gas and placed in sterilized vermiculite to germinate. Ten days after germination, 15 mL of a rhizobial suspension (OD_600nm_ = 0.08) of *B. japonicum* USDA110 was poured over the seeds. Nodule evaluation was conducted 21 days after inoculation (dai). All soybean plants used for nodule phenotyping were cultivated under low‐nitrate conditions and irrigated with a nitrogen‐free nutrient solution (Li *et al*., 2018).

### Vector construction and plant transformation

cDNA template used for gene cloning was derived from c.v. Wm82. The target sequence for *INN1* gene editing was designed using CRISPR direct (http://crispr.dbcls.jp/) (Naito *et al*., 2015). The target sequence was subcloned into various single‐guide RNA (sgRNA) expression cassettes and subsequently transferred into the pYLCRISPR/Cas9‐DB vector (Ma *et al*., 2015). The construct was introduced into *Agrobacterium tumefaciens* GV3101. For soybean stable transformation, the cotyledon‐node method was used as described previously (Flores *et al*., 2008). The IN*N1* overexpression lines are described previously (Zhao *et al*., 2024).

For transient dual‐luciferase reporter assays, the coding sequences of *INN1*, *LUX1* and *LUX2* were inserted between the *Mul*I and *Spe*I restriction sites in the pTF101‐GFP vector and driven by the Cauliflower mosaic virus (CaMV) *35S* promoter. For construction of the *ENOD40*_*pro*_ *:LUC* reporter vector, a 2‐kb promoter fragment was inserted between the *Kpn*I and *Bam*HI restriction sites in pGreen0800 and fused with the *LUCIFERASE* (*LUC*) reporter gene.

For RNAi vector construction, the *LUX* RNAi construct includes a 213‐bp *LUX1* coding‐region segment that inserted in the sense and antisense directions into the pG2RNAi2 vector through *Asc*I/*Swa*I and *Bam*HI/*Avr*II sites, respectively. The *ENOD40* RNAi construct contains a 305‐bp *ENOD40* coding region inserted into the pG2RNAi2 vector between *Asc*I/*Swa*I and *Bam*HI/*Avr*II sites.

For the construction of the promoter:*GUS* reporter constructs *INN1*_*pro*_ *:GUS*, *LUX1*_*pro*_ *:GUS* and *LUX2*_*pro*_ *:GUS*, approximately 2‐kb promoter sequences were inserted between the *Xba*I and *Bam*HI restriction sites of the GUS vector pUBI‐GFP‐GUS to drive expression of *GUS*.

All primers used for plasmid construction are listed in Table S1.

### Hairy roots transformation

To obtain transgenic composite *G. max* plants, RNAi or *GUS* expression constructs were transformed into *A. rhizogenes* K599 with modifications based on previously described methods (Kereszt *et al*., 2007). *Agrobacteria* containing the desired vector were cultured in LB medium with appropriate antibiotic selection to OD_600nm_ of 0.8, after which cells were resuspended in CCM medium. Five‐day‐old soybean seedlings were selected, and cotyledon tips were excised approximately 1 cm below the base, with a cross‐cut made at the incision. The wounded end was immersed in the bacterial suspension in CCM medium for 30 min. Explants were then transferred to Petri dishes containing moistened filter paper and cultured in darkness at 28 °C for 2–3 days. Following this, explants were transferred to sterilized vermiculite for root formation and covered to maintain humidity. After 10 days of culture in a high‐humidity environment, GFP fluorescence was detected using a handheld fluorescence lamp (Luyor 3415 RG; Luyor, China) to confirm transformation.

For nodule phenotyping, *B. japonicum* USDA110 was inoculated at OD_600nm_ = 0.08 in 30 mL of YMA culture medium, and the nodule phenotype was assessed 28 days later. For GUS staining, positive roots inoculated with rhizobia at different time points were selected, and histochemical GUS staining was performed in accordance with a published protocol (Li *et al*., 2018).

### RNA extraction and real‐time quantitative PCR (RT–qPCR)

Total RNA was extracted from various tissues using the Vazyme FastPure Universal Plant Total RNA Isolation Kit, following the manufacturer's instructions. Next, 1 μg of total RNA was reverse transcribed into cDNA using the HiScript III RT SuperMix for qPCR (+gDNA wiper) according to the manufacturer's guidelines. RT–qPCR was performed using ChamQ Universal SYBR qPCR Master Mix with a two‐step program: first at 94 °C for 1 min, followed by 45 cycles of 94 °C for 10 s, 60 °C for 30 s and 72 °C for 10 s, with a final dissociation‐curve stage. Expression was normalized to *ACTIN11* (*Glyma.18G290800*) and relative expression was calculated using the 2^−ΔΔCT^ equation (Livak and Schmittgen, 2001). Primers used for RT–qPCR are listed in Table S1.

### RNA‐seq and data analysis

Seeds of Wm82 and *inn1‐2* were sterilized using chlorine gas and then placed on sterilized vermiculite for germination. After 10 days, seedlings were irrigated with 15 mL of *B. japonicum* USDA110 suspension at OD_600nm_ = 0.08, mock inoculation 15 mL of sterile water. Three days later, soybean roots were collected at ZT 12. Total RNA extraction was performed as described above. We performed sequence by Biomarker Technologies Co., Ltd (Beijing, China). Clean Reads were rapidly and accurately aligned to the reference genome using HISAT2 software to obtain the mapping information of reads on the reference genome (Kim *et al*., 2015). Then, StringTie was used to assemble the aligned reads (Pertea *et al*., 2015). Transcript abundance was calculated using FPKM (fragments perkilobase of exon per million fragments mapped). DEGs were identified based on an absolute fold change of ≥2 and FDR <0.01. KEGG analysis (https://yanglab.hzau.edu.cn/SoyMD/#/tools/kegg) was performed on the DEGs. *P* < 0.05 was considered statistically significantly enriched in pathways.

### ChIP–qPCR

Transgenic roots expressing proteins fused to 6 × HA or FLAG tags were collected for ChIP assays 3 days post‐inoculation with *Bradyrhizobium japonicum* strain USDA110, with non‐inoculated roots serving as the control group. Root samples were thoroughly washed and placed in Tris–HCl buffer (pH 8.0) containing 1% (v/v) formaldehyde for vacuum infiltration at 85 kPa for 5 min, followed by slow depressurization. This crosslinking procedure was repeated 3–4 times. To quench the crosslinking reaction, 2.5 M glycine was added, and the samples were washed 2–3 times with ddH_2_O before being stored at −80 °C.

For chromatin extraction, 0.5 g of fixed root tissue was ground under low‐temperature conditions and resuspended in 10 mL of Extraction Buffer I (10 mM Tris–HCl (pH 8.0), 1 mM EDTA, 0.25 M sucrose, 10 mM KCl, 40 mM NaCl, 0.1% SDS, 1% Triton X‐100, 1 mM PMSF, 1× protease inhibitor cocktail (PI)). The suspension was incubated at 4 °C with rotation for 5 min, filtered through a 40‐μm filter, and centrifuged at 6000 rcf, 4 °C for 5 min. The pellet was resuspended in 1 mL of Extraction Buffer II (10 mM Tris–HCl (pH 8.0), 1 mM EDTA, 0.1% SDS, 1% Triton X‐100, 1 mM PMSF, 1× PI) and centrifuged again at 6000 rcf, 4 °C for 5 min. The resulting pellet was then resuspended in 300 μL of Nuclei Lysis Buffer (50 mM Tris–HCl (pH 8.0), 5 mM EDTA, 0.5% SDS, 1 mM PMSF, 1× PI).

Chromatin fragmentation was performed using a Diagenode Bioruptor UCD 300 non‐contact ultrasonic homogenizer at 4 °C, with the following settings: 10 s ON, 30 s OFF, for 20 cycles. The fragmented chromatin was precleared by incubating with Protein A/G magnetic beads (Sigma‐Aldrich) at 4 °C for 30–60 min with rotation to reduce nonspecific binding. Immunoprecipitation was performed by incubating the precleared chromatin with anti‐FLAG or anti‐HA antibodies overnight at 4 °C, followed by capture with Protein A/G magnetic beads (Sigma‐Aldrich). After extensive washing with low‐salt and high‐salt buffers, immune complexes were eluted and subjected to reverse crosslinking.

DNA was then purified using the Zymo DNA Purification Kit (Cat. No. D4003) according to the manufacturer's protocol. qPCR assays were performed using primers targeting different regions of the *ENOD40* promoter, with *ELF1b* serving as the internal control. Primer sequences are provided in Table S1.

### ChIP‐seq

Following chromatin immunoprecipitation (ChIP) and DNA purification, ChIP‐seq library preparation was performed using the Vazyme TD501 Hyper Library Construction Kit, following the manufacturer's instructions with slight modifications. Tagmentation was conducted on Protein A/G bead‐bound ChIP DNA using Tn5 transposase at 37 °C for 30 min with continuous rotation. The beads were then washed sequentially with low‐salt, high‐salt and TE buffers to remove excess transposase.

After reverse crosslinking and protein digestion, DNA was purified using the Zymo DNA Purification Kit (Cat. No. D4003) and quantified using a Qubit Fluorometer (Thermo Fisher). Library amplification was performed using Q5 polymerase, starting with an initial 10‐cycle pre‐amplification, followed by qPCR quantification using ChamQ Universal SYBR qPCR Master Mix to determine the optimal number of additional cycles (Ct + 5–10 cycles). Final library amplification was performed under standard conditions, and libraries were purified using AMPure XP beads (Beckman Coulter). The purified library was quantified using Qubit, and fragment size distribution was assessed using an Agilent Bioanalyzer or TapeStation. Sequencing was performed on an Illumina platform by Novogene Biotechnology Co., Ltd., using paired‐end 150 bp (PE150) or single‐end 50 bp (SE50) reads, depending on the experimental design.

### EMSA

EMSA analysis was performed using the LightShift Chemiluminescent EMSA Kit (Pierce) according to the manufacturer's instructions and the method described by Yoo *et al*. (2010). First, MBP‐tagged LUX1, LUX2 or INN1 proteins were expressed in *E. coli* BL21 cells (Lin *et al*., 2022). The cells are cultured until the OD600 reaches 0.6–0.8, then 1 mM IPTG is added to induce protein expression at 16 °C for 16 h. The lysate is clarified by centrifugation, and the soluble protein fraction is collected. Next, primers containing LUX binding sites and biotin labels were synthesized by Qingke Company and annealed. The following components were sequentially added to the reaction mixture: probe, protein and binding buffer. The mixture was gently mixed and incubated at room temperature for 20 min. After separating the protein–probe complexes using a 6% non‐denaturing polyacrylamide gel, samples were transferred onto a nylon membrane. The membrane was crosslinked face‐up for 2–3 min under 312‐nm UV light. It was then incubated in blocking buffer for 10 min. Streptavidin–HRP conjugate was added and incubated for 20 min, followed by three washes of the membrane with washing buffer, 10 min each time. Finally, the membrane was incubated with the supplied substrate solution at room temperature for 5 min and exposed for chemiluminescent detection.

### Transient dual‐luciferase reporter assays

The *ENOD40*_*pro*_ *:LUC* construct was used as the reporter cassette, with GFP, INN1–GFP, LUX1–GFP and LUX2–GFP vectors (all in the pTF‐GFP vector backbone) serving as effectors. These vectors were individually transformed into *A. tumefaciens* GV3101 and infiltration of *Nicotiana benthamiana* was performed following Hu *et al*. (2013). After 3 days, relative activities of firefly luciferase (LUC) and Renilla luciferase (REN) were analysed using the Firefly Luciferase Reporter Gene Assay Kit (Beyotime, China) and detected on a Biotek Synergy H1 microplate reader (Agilent, USA). The experiment was conducted with five independent biological replicates.

### Statistical analyses

Statistical analysis was performed using Microsoft Excel (ver. 2017) and GraphPad Prism 8 (ver. 8.0.2). ANOVA, Student's *t*‐tests, Kruskal–Wallis test and Duncan's multiple comparison test were conducted to calculate *P*‐values.

## Conflict of interest

The authors declare no competing interests.

## Author contributions

F. Kong designed and supervised the experiments and managed the projects. B. Su, Z. Sun, Ho. Li, Ha. Li, K. Zhang, C. Fan, M. Zhong, H. Zou, R. Li, L. Chen, M. Huang performed the experiment. J. Jin provided mutant material. B. Su, Z. Sun and Ho. Li performed the data analysis. B. Su drafted the manuscript. Z. Sun, B. Liu, F. Kong revised the manuscript. All authors have read and approved the final version of the manuscript.

## Supporting information


**Figure S1** Distribution of root‐nodule numbers in individual plants from the F_2_ segregating population derived from the cross between XL1 and the *inn1* mutant.
**Figure S2** BSA mapping of the *INN1* gene.
**Figure S3** Nodule phenotypes of the F_2_ segregating population derived from the cross between Wm82 and *inn1*.
**Figure S4** Genetic mapping of the *INN1* gene.
**Figure S5** Generation and characterization of *INN1* mutants and transgenic overexpression lines.
**Figure S6** *INN1*, *LUX1* and *LUX2* are all expressed in several soybean tissues.
**Figure S7** Identification of LUX Binding Sites via ChIP‐seq Data Analysis.
**Figure S8** INN1–MBP does not directly bind to the ENOD40 promoter in the EMSA.
**Figure S9** *ENOD40* expression levels in different transgenic hairy root lines.
**Figure S10** *ENOD40* expression levels in different soybean genotypes.
**Figure S11** Characteristics of mature plants of XL1 and *inn1* mutant.
**Figure S12** The INN1–LUX complex does not bind to the *ENOD40* promoter in the absence of rhizobial inoculation.

**Table S1** List of primers used in this study.
**Table S2** Difference SNPs/indels within the candidate interval.
**Table S3** High‐Quality SNPs after filtering.
**Table S4** High‐Quality InDel after filtering.
**Table S5** Clean data statistics.
**Table S6** Reference sequence alignment results.
**Table S7** Annotation of ChIP‐seq peaks in root of soybean.
**Table S8** KEGG pathway analysis of genes overlapping between ChIP‐seq and RNA‐seq data.
**Table S9** Significantly differentially expressed genes in the root after USDA110 treatment.
**Table S10** ChIP‐seq traces the chromatin state of target genes.

## Data Availability

The data that supports the findings of this study are available in the supplementary material of this article.

## References

[pbi70053-bib-0001] Andrade, L. , Lu, Y. , Cordeiro, A. , Costa, J.M.F. , Wigge, P.A. , Saibo, N.J.M. and Jaeger, K.E. (2022) The evening complex integrates photoperiod signals to control flowering in rice. Proc. Natl. Acad. Sci. USA 119, e2122582119. 35733265 10.1073/pnas.2122582119 PMC9245669

[pbi70053-bib-0002] Bu, T. , Lu, S. , Wang, K. , Dong, L. , Li, S. , Xie, Q. , Xu, X. *et al*. (2021) A critical role of the soybean evening complex in the control of photoperiod sensitivity and adaptation. Proc. Natl. Acad. Sci. USA 118(8), e2010241118. 33558416 10.1073/pnas.2010241118 PMC7923351

[pbi70053-bib-0003] Campalans, A. , Kondorosi, A. and Crespi, M. (2004) *Enod40*, a short open reading frame‐containing mRNA, induces cytoplasmic localization of a nuclear RNA binding protein in *Medicago truncatula* . Plant Cell 16, 1047–1059. 15037734 10.1105/tpc.019406 PMC412876

[pbi70053-bib-0004] Carroll, B.J. , McNeil, D.L. and Gresshoff, P.M. (1985) A supernodulation and *nitrate‐tolerant symbiotic* (*nts*) soybean mutant. Plant Physiol. 78, 34–40. 16664203 10.1104/pp.78.1.34 PMC1064671

[pbi70053-bib-0005] Charon, C. , Johansson, C. , Kondorosi, E. , Kondorosi, A. and Crespi, M. (1997) *ENOD40* induces dedifferentiation and division of root cortical cells in legumes. Proc. Natl. Acad. Sci. USA 94, 8901–8906. 11038563 10.1073/pnas.94.16.8901 PMC23190

[pbi70053-bib-0006] Charon, C. , Sousa, C. , Crespi, M. and Kondorosi, A. (1999) Alteration of *enod40* expression modifies medicago truncatula root nodule development induced by *sinorhizobium meliloti* . Plant Cell 11, 1953–1966. 10521525 10.1105/tpc.11.10.1953 PMC144109

[pbi70053-bib-0007] Cheng, Q. , Gan, Z. , Wang, Y. , Lu, S. , Hou, Z. , Li, H. , Xiang, H. *et al*. (2020) The soybean gene *J* contributes to salt stress tolerance by up‐regulating salt‐responsive genes. Front. Plant Sci. 11, 272. 32256507 10.3389/fpls.2020.00272 PMC7090219

[pbi70053-bib-0008] Dong, L. , Fang, C. , Cheng, Q. , Su, T. , Kou, K. , Kong, L. , Zhang, C. *et al*. (2021a) Genetic basis and adaptation trajectory of soybean from its temperate origin to tropics. Nat. Commun. 12, 5445. 34521854 10.1038/s41467-021-25800-3 PMC8440769

[pbi70053-bib-0009] Dong, W. , Zhu, Y. , Chang, H. , Wang, C. , Yang, J. , Shi, J. , Gao, J. *et al*. (2021b) An SHR–SCR module specifies legume cortical cell fate to enable nodulation. Nature 589, 586–590. 33299183 10.1038/s41586-020-3016-z

[pbi70053-bib-0010] Downie, J.A. (2014) Legume nodulation. Curr. Biol. 24, R184–R190. 24602880 10.1016/j.cub.2014.01.028

[pbi70053-bib-0011] Endre, G. , Kereszt, A. , Kevei, Z. , Mihacea, S. , Kaló, P. and Kiss, G.B. (2002) A receptor kinase gene regulating symbiotic nodule development. Nature 417, 962–966. 12087406 10.1038/nature00842

[pbi70053-bib-0012] Erisman, J.W. , Galloway, J.N. , Seitzinger, S.P. , Bleeker, A. and Butterbach‐Bahl, K. (2011) Reactive nitrogen in the environment and its effect on climate change. Curr. Opin. Environ. Sustain. 3, 281–290.

[pbi70053-bib-0013] Fang, X. , Han, Y. , Liu, M. , Jiang, J. , Li, X. , Lian, Q. , Xie, X. *et al*. (2021) Modulation of evening complex activity enables north‐to‐south adaptation of soybean. Sci. China Life Sci. 64, 179–195. 33230598 10.1007/s11427-020-1832-2

[pbi70053-bib-0014] Flores, T. , Karpova, O. , Su, X. , Zeng, P. , Bilyeu, K. , Sleper, D.A. , Nguyen, H.T. *et al*. (2008) Silencing of *GmFAD3* gene by siRNA leads to low alpha‐linolenic acids (18:3) of *fad3*‐mutant phenotype in soybean [*Glycine max* (Merr.)]. Transgenic Res. 17, 839–850. 18256901 10.1007/s11248-008-9167-6

[pbi70053-bib-0015] Gu, B. , Zhang, X. , Lam, S.K. , Yu, Y. , van Grinsven, H.J.M. , Zhang, S. , Wang, X. *et al*. (2023) Cost‐effective mitigation of nitrogen pollution from global croplands. Nature 613(7942), 77–84. 36600068 10.1038/s41586-022-05481-8 PMC9842502

[pbi70053-bib-0067] Helfer, A. , Nusinow, D.A. , Chow, B.Y. , Gehrke, A.R. , Bulyk, M.L. and Kay, S.A. (2011) LUX ARRHYTHMO encodes a nighttime repressor of circadian gene expression in the Arabidopsis core clock. Curr. Biol 21, 126‐133. 21236673 10.1016/j.cub.2010.12.021 PMC3057456

[pbi70053-bib-0016] Herridge, D.F. , Peoples, M.B. and Boddey, R.M. (2008) Global inputs of biological nitrogen fixation in agricultural systems. Plant Sci. 311, 1–18.

[pbi70053-bib-0017] Hill, J.T. , Demarest, B.L. , Bisgrove, B.W. , Gorsi, B. , Su, Y.C. and Yost, H.J. (2013) MMAPPR: mutation mapping analysis pipeline for pooled RNA‐seq. Genome Res. 23, 687–697. 23299975 10.1101/gr.146936.112 PMC3613585

[pbi70053-bib-0018] Hu, Y. , Chen, L. , Wang, H. , Zhang, L. , Wang, F. and Yu, D. (2013) *Arabidopsis* transcription factor *WRKY8* functions antagonistically with its interacting partner VQ9 to modulate salinity stress tolerance. Plant J. 74, 730–745. 23451802 10.1111/tpj.12159

[pbi70053-bib-0019] Jung, J.‐H. , Barbosa, A.D. , Hutin, S. , Kumita, J.R. , Gao, M. , Derwort, D. , Silva, C.S. *et al*. (2020) A prion‐like domain in ELF3 functions as a thermosensor in *Arabidopsis* . Nature 585(7824), 256–260. 32848244 10.1038/s41586-020-2644-7

[pbi70053-bib-0020] Kereszt, A. , Li, D. , Indrasumunar, A. , Nguyen, C.D. , Nontachaiyapoom, S. , Kinkema, M. and Gresshoff, P.M. (2007) Agrobacterium rhizogenes‐mediated transformation of soybean to study root biology. Nat. Protoc. 2, 948–952. 17446894 10.1038/nprot.2007.141

[pbi70053-bib-0021] Kim, D. , Langmead, B. and Salzberg, S.L. (2015) HISAT: a fast spliced aligner with low memory requirements. Nat. Methods 12, 357–360. 25751142 10.1038/nmeth.3317 PMC4655817

[pbi70053-bib-0022] Kong, Y. , Zhang, Y. , Liu, X. , Meng, Z. , Yu, X. , Zhou, C. and Han, L. (2022) The conserved and specific roles of the *LUX ARRHYTHMO* in circadian clock and nodulation. Int. J. Mol. Sci. 23, 3473. 35408833 10.3390/ijms23073473 PMC8998424

[pbi70053-bib-0023] Kumagai, H. , Kinoshita, E. , Ridge, R.W. and Kouchi, H. (2006) RNAi knock‐down of *ENOD40s* leads to significant suppression of nodule formation in *Lotus japonicus* . Plant Cell Physiol. 47, 1102–1111. 16816411 10.1093/pcp/pcj081

[pbi70053-bib-0024] Li, X. , Zheng, J. , Yang, Y. and Liao, H. (2018) *INCREASING NODULE SIZE1* expression is required for normal rhizobial symbiosis and nodule development. Plant Physiol. 178, 1233–1248. 30266750 10.1104/pp.18.01018 PMC6236598

[pbi70053-bib-0025] Li, H. , Chen, Z. , Wang, F. , Xiang, H. , Liu, S. , Gou, C. , Fang, C. *et al*. (2024) *J*‐family genes redundantly regulate flowering time and increase yield in soybean. Crop J 12, 944–949.

[pbi70053-bib-0026] Limpens, E. , Franken, C. , Smit, P. , Willemse, J. , Bisseling, T. and Geurts, R. (2003) LysM domain receptor kinases regulating rhizobial Nod factor‐induced infection. Science 302, 630–633. 12947035 10.1126/science.1090074

[pbi70053-bib-0027] Lin, X. , Dong, L. , Tang, Y. , Li, H. , Cheng, Q. , Li, H. , Zhang, T. *et al*. (2022) Novel and multifaceted regulations of photoperiodic flowering by phytochrome A in soybean. Proc. Natl. Acad. Sci. USA 119, e2208708119. 36191205 10.1073/pnas.2208708119 PMC9565047

[pbi70053-bib-0028] Liu, C.W. , Breakspear, A. , Guan, D. , Cerri, M.R. , Jackson, K. , Jiang, S. , Robson, F. *et al*. (2019) NIN acts as a network hub controlling a growth module required for rhizobial infection. Plant Physiol. 179, 1704–1722. 30710053 10.1104/pp.18.01572 PMC6446755

[pbi70053-bib-0029] Livak, K.J. and Schmittgen, T.D. (2001) Analysis of relative gene expression data using real‐time quantitative PCR and the 2(‐Delta Delta C(T)) method. Methods 25, 402–408. 11846609 10.1006/meth.2001.1262

[pbi70053-bib-0030] Lu, S. , Zhao, X. , Hu, Y. , Liu, S. , Nan, H. , Li, X. , Fang, C. *et al*. (2017) Natural variation at the soybean *J* locus improves adaptation to the tropics and enhances yield. Nat. Genet. 49, 773–779. 28319089 10.1038/ng.3819

[pbi70053-bib-0031] Ma, X. , Zhang, Q. , Zhu, Q. , Liu, W. , Chen, Y. , Qiu, R. , Wang, B. *et al*. (2015) A robust CRISPR/Cas9 system for convenient, high‐efficiency multiplex genome editing in monocot and dicot plants. Mol. Plant 8, 1274–1284. 25917172 10.1016/j.molp.2015.04.007

[pbi70053-bib-0032] Ma, W. , Liu, W. , Hou, W. , Sun, S. , Jiang, B. , Han, T. , Feng, Y. *et al*. (2019) *GmNMH7*, a MADS‐box transcription factor, inhibits root development and nodulation of soybean (*Glycine max* [L.] Merr.). J. Integr. Agric. 18, 553–562.

[pbi70053-bib-0033] Madsen, E.B. , Madsen, L.H. , Radutoiu, S. , Olbryt, M. , Rakwalska, M. , Szczyglowski, K. , Sato, S. *et al*. (2003) A receptor kinase gene of the LysM type is involved in legume perception of rhizobial signals. Nature 425(6958), 637–640. 14534591 10.1038/nature02045

[pbi70053-bib-0034] Marsh, J.F. , Rakocevic, A. , Mitra, R.M. , Brocard, L. , Sun, J. , Eschstruth, A. , Long, S.R. *et al*. (2007) *Medicago truncatula NIN* is essential for rhizobial‐independent nodule organogenesis induced by autoactive calcium/calmodulin‐dependent protein kinase. Plant Physiol. 144, 324–335. 17369436 10.1104/pp.106.093021 PMC1913781

[pbi70053-bib-0035] Meng, L. , Li, H. , Zhang, L. and Wang, J. (2015) QTL IciMapping: integrated software for genetic linkage map construction and quantitative trait locus mapping in biparental populations. Crop J. 3, 269–283.

[pbi70053-bib-0036] Naito, Y. , Hino, K. , Bono, H. and Ui‐Tei, K. (2015) CRISPRdirect: software for designing CRISPR/Cas guide RNA with reduced off‐target sites. Bioinformatics 31, 1120–1123. 25414360 10.1093/bioinformatics/btu743 PMC4382898

[pbi70053-bib-0037] Nap, J.P. and Bisseling, T. (1990) Developmental biology of a plant‐prokaryote symbiosis: the legume root nodule. Science 250, 948–954. 17746918 10.1126/science.250.4983.948

[pbi70053-bib-0038] Nishida, H. and Suzaki, T. (2018) Nitrate‐mediated control of root nodule symbiosis. Curr. Opin. Plant Biol. 44, 129–136. 29684704 10.1016/j.pbi.2018.04.006

[pbi70053-bib-0039] Nleya, T. , Sexton, P. , Gustafson, K. and Miller, J.M. (2013) Soybean growth stages. IGrow soybean: Best Management Practices for soybean production.

[pbi70053-bib-0040] Nusinow, D.A. , Helfer, A. , Hamilton, E.E. , King, J.J. , Imaizumi, T. , Schultz, T.F. , Farré, E.M. *et al*. (2011) The ELF4‐ELF3‐LUX complex links the circadian clock to diurnal control of hypocotyl growth. Nature 475, 398–402. 21753751 10.1038/nature10182 PMC3155984

[pbi70053-bib-0041] Oldroyd, G.E.D. (2013) Speak, friend, and enter: signalling systems that promote beneficial symbiotic associations in plants. Nat. Rev. Microbiol. 11, 252–263. 23493145 10.1038/nrmicro2990

[pbi70053-bib-0042] Peck, M.C. , Fisher, R.F. and Long, S.R. (2006) Diverse flavonoids stimulate NodD1 binding to *nod* gene promoters in *Sinorhizobium meliloti* . J. Bacteriol. 188, 5417–5427. 16855231 10.1128/JB.00376-06 PMC1540014

[pbi70053-bib-0043] Pertea, M. , Pertea, G.M. , Antonescu, C.M. , Chang, T.C. , Mendell, J.T. and Salzberg, S.L. (2015) StringTie enables improved reconstruction of a transcriptome from RNA‐seq reads. Nat. Biotechnol. 33, 290–295. 25690850 10.1038/nbt.3122 PMC4643835

[pbi70053-bib-0044] Poffenbarger, H.J. , Barker, D.W. , Helmers, M.J. , Miguez, F.E. , Olk, D.C. , Sawyer, J.E. , Six, J. *et al*. (2017) Maximum soil organic carbon storage in Midwest U.S. cropping systems when crops are optimally nitrogen‐fertilized. PLoS One 12, e0172293. 28249014 10.1371/journal.pone.0172293 PMC5332021

[pbi70053-bib-0045] Qin, C. , Li, H. , Zhang, S. , Lin, X. , Jia, Z. , Zhao, F. , Wei, X. *et al*. (2023) GmEID1 modulates light signaling through the Evening Complex to control flowering time and yield in soybean. Proc. Natl. Acad. Sci. USA 120, e2212468120. 37011215 10.1073/pnas.2212468120 PMC10104576

[pbi70053-bib-0046] Radutoiu, S. , Madsen, L.H. , Madsen, E.B. , Felle, H.H. , Umehara, Y. , Grønlund, M. , Sato, S. *et al*. (2003) Plant recognition of symbiotic bacteria requires two LysM receptor‐like kinases. Nature 425, 585–592. 14534578 10.1038/nature02039

[pbi70053-bib-0047] Rohrig, H. , Schmidt, J. , Miklashevichs, E. , Schell, J. and John, M. (2002) Soybean *ENOD40* encodes two peptides that bind to sucrose synthase. Proc. Natl. Acad. Sci. USA 99, 1915–1920. 11842184 10.1073/pnas.022664799 PMC122294

[pbi70053-bib-0048] Rubenach, A.J.S. , Hecht, V. , Vander Schoor, J.K. , Liew, L.C. , Aubert, G. , Burstin, J. and Weller, J.L. (2017) EARLY FLOWERING3 redundancy fine‐tunes photoperiod sensitivity. Plant Physiol. 173, 2253–2264. 28202598 10.1104/pp.16.01738 PMC5373058

[pbi70053-bib-0049] Sakuraba, Y. , Bülbül, S. , Piao, W. , Choi, G. and Paek, N.‐C. (2017) Arabidopsis *EARLY FLOWERING3* increases salt tolerance by suppressing salt stress response pathways. Plant J. 92, 1106–1120. 29032592 10.1111/tpj.13747

[pbi70053-bib-0050] Schiessl, K. , Lilley, J.L.S. , Lee, T. , Tamvakis, I. , Kohlen, W. , Bailey, P.C. , Thomas, A. *et al*. (2019) *NODULE INCEPTION* recruits the lateral root developmental program for symbiotic nodule organogenesis in *Medicago truncatula* . Curr. Biol. 29, 3657–3668. 31543454 10.1016/j.cub.2019.09.005 PMC6839406

[pbi70053-bib-0051] Shrestha, A. , Zhong, S. , Therrien, J. , Huebert, T. , Sato, S. , Mun, T. , Andersen, S.U. *et al*. (2021) *Lotus japonicus Nuclear Factor YA1*, a nodule emergence stage‐specific regulator of auxin signalling. New Phytol. 229, 1535–1552. 32978812 10.1111/nph.16950 PMC7984406

[pbi70053-bib-0052] Smit, P. , Limpens, E. , Geurts, R. , Fedorova, E. , Dolgikh, E. , Gough, C. and Bisseling, T. (2007) Medicago LYK3, an entry receptor in rhizobial nodulation factor signaling. Plant Physiol. 145, 183–191. 17586690 10.1104/pp.107.100495 PMC1976573

[pbi70053-bib-0053] Soyano, T. , Shimoda, Y. , Kawaguchi, M. and Hayashi, M. (2019) A shared gene drives lateral root development and root nodule symbiosis pathways in *Lotus* . Science 366, 1021–1023. 31754003 10.1126/science.aax2153

[pbi70053-bib-0054] Tilman, D. (1999) Global environmental impacts of agricultural expansion: the need for sustainable and efficient practices. Proc. Natl. Acad. Sci. USA 96, 5995–6000. 10339530 10.1073/pnas.96.11.5995 PMC34218

[pbi70053-bib-0055] Xie, Y. , Zhao, Y. , Chen, L. , Wang, Y. , Xue, W. , Kong, D. , Li, C. *et al*. (2024) ZmELF3.1 integrates the RA2‐TSH4 module to repress maize tassel branching. New Phytol. 241, 490–503. 37858961 10.1111/nph.19329

[pbi70053-bib-0056] Xu, H. , Li, Y. , Zhang, K. , Li, M. , Fu, S. , Tian, Y. , Qin, T. *et al*. (2021) miR169c‐NFYA‐C‐ENOD40 modulates nitrogen inhibitory effects in soybean nodulation. New Phytol. 229(6), 3377–3392. 33245793 10.1111/nph.17115

[pbi70053-bib-0057] Yang, W.C. , Katinakis, P. , Hendriks, P. , Smolders, A. , de Vries, F. , Spee, J. , van Kammen, A. *et al*. (1993) Characterization of *GmENOD40*, a gene showing novel patterns of cell‐specific expression during soybean nodule development. Plant J. 3, 573–585. 8220464 10.1046/j.1365-313x.1993.03040573.x

[pbi70053-bib-0058] Yang, J. , Lan, L. , Jin, Y. , Yu, N. , Wang, D. and Wang, E. (2022) Mechanisms underlying legume‐rhizobium symbioses. J. Integr. Plant Biol. 64, 244–267. 34962095 10.1111/jipb.13207

[pbi70053-bib-0059] Yoo, C.Y. , Pence, H.E. , Jin, J.B. , Miura, K. , Gosney, M.J. , Hasegawa, P.M. and Mickelbart, M.V. (2010) The *Arabidopsis* GTL1 transcription factor regulates water use efficiency and drought tolerance by modulating stomatal density via transrepression of *SDD1* . Plant Cell 22, 4128–4141. 21169508 10.1105/tpc.110.078691 PMC3027182

[pbi70053-bib-0060] Yue, Y. , Liu, N. , Jiang, B. , Li, M. , Wang, H. , Jiang, Z. , Pan, H. *et al*. (2017) A single nucleotide deletion in *J* encoding GmELF3 confers long juvenility and is associated with adaption of tropic soybean. Mol. Plant 10, 656–658. 27979775 10.1016/j.molp.2016.12.004

[pbi70053-bib-0061] Yun, J. , Sun, Z. , Jiang, Q. , Wang, Y. , Wang, C. , Luo, Y. , Zhang, F. *et al*. (2022) The miR156b‐GmSPL9d module modulates nodulation by targeting multiple core nodulation genes in soybean. New Phytol. 233, 1881–1899. 34862970 10.1111/nph.17899 PMC9303946

[pbi70053-bib-0062] Yun, J. , Wang, C. , Zhang, F. , Chen, L. , Sun, Z. , Cai, Y. , Luo, Y. *et al*. (2023) A nitrogen fixing symbiosis‐specific pathway required for legume flowering. Sci. Adv. 9, eade1150. 36638166 10.1126/sciadv.ade1150 PMC9839322

[pbi70053-bib-0063] Zagotta, M.T. , Hicks, K.A. , Jacobs, C.I. , Young, J.C. , Hangarter, R.P. and Meeks‐Wagner, D.R. (1996) The *Arabidopsis ELF3* gene regulates vegetative photomorphogenesis and the photoperiodic induction of flowering. Plant J. 10, 691–702. 8893545 10.1046/j.1365-313x.1996.10040691.x

[pbi70053-bib-0064] Zhang, Y. , Cheng, Q. , Liao, C. , Li, L. , Gou, C. , Chen, Z. , Wang, Y. *et al*. (2022) GmTOC1b inhibits nodulation by repressing *GmNIN2a* and *GmENOD40‐1* in soybean. Front. Plant Sci. 13, 1052017. 36438085 10.3389/fpls.2022.1052017 PMC9691777

[pbi70053-bib-0065] Zhao, X. , Li, H. , Wang, L. , Wang, J. , Huang, Z. , Du, H. , Li, Y. *et al*. (2024) A critical suppression feedback loop determines soybean photoperiod sensitivity. Dev. Cell 59, 1750–1763.e1754. 38688276 10.1016/j.devcel.2024.04.004

[pbi70053-bib-0066] Zhong, X. , Wang, J. , Shi, X. , Bai, M. , Yuan, C. , Cai, C. , Wang, N. *et al*. (2024) Genetically optimizing soybean nodulation improves yield and protein content. Nat. Plants 10, 736–742. 38724696 10.1038/s41477-024-01696-x

