# Supplementary material for: Evening complex component ELF3 interacts with LUX proteins to repress soybean root nodulation
Source: Plant Biotechnol J. 2025 Mar 17;23(6):2194–206. doi: 10.1111/pbi.70053 (PMC12120873; doi:10.1111/pbi.70053)
Supplement: Supplementary file 1 — Figure S1 Distribution of root‐nodule numbers in individual plants from the F2 segregating population derived from the cross between XL1 and the inn1 mutant. Figure S2 BSA mapping of the INN1 gene. Figure S3 Nodule phenotypes of the F2 segregating population derived from the cross between Wm82 and inn1. Figure S4 Genetic mapping of the INN1 gene. Figure S5 Generation and characterization of INN1 mutants and transgenic overexpression lines. Figure S6 INN1, LUX1 and LUX2 are all expressed in several soybean tissues. Figure S7 Identification of LUX Binding Sites via ChIP‐seq Data Analysis. Figure S8 INN1–MBP does not directly bind to the ENOD40 promoter in the EMSA. Figure S9 ENOD40 expression levels in different transgenic hairy root lines. Figure S10 ENOD40 expression levels in different soybean genotypes. Figure S11 Characteristics of mature plants of XL1 and inn1 mutant. Figure S12 The INN1–LUX complex does not bind to the ENOD40 promoter in the absence of rhizobial inoculation. [file PBI-23-2194-s001.docx]

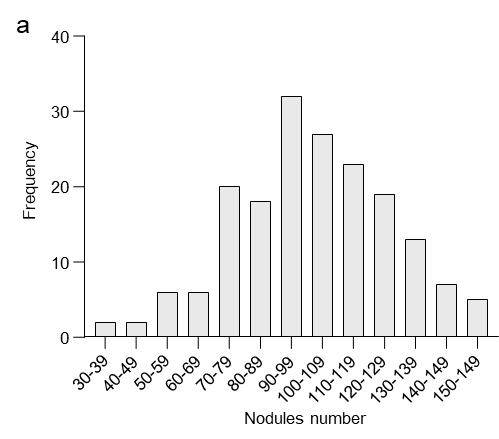


**Fig. S1. Distribution of root-nodule numbers in individual plants from the F_2_ segregating population derived from the cross between XL1 and the *inn1* mutant.**


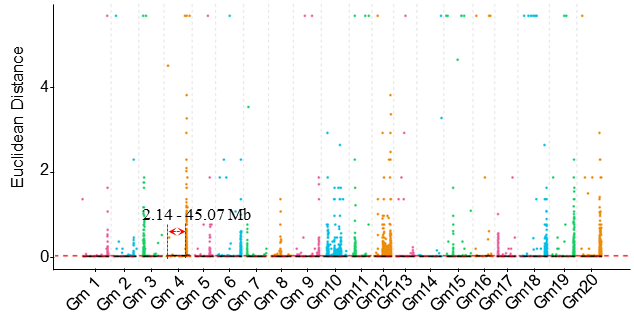


**Fig. S2. BSA mapping of the *INN1* gene.**

The x-axis represents chromosome numbers, with colored dots indicating the Euclidean Distance (ED) values of each SNP site. The black line represents the fitted ED values, and the red dashed line indicates the statistical significance threshold (*P* = 0.01).


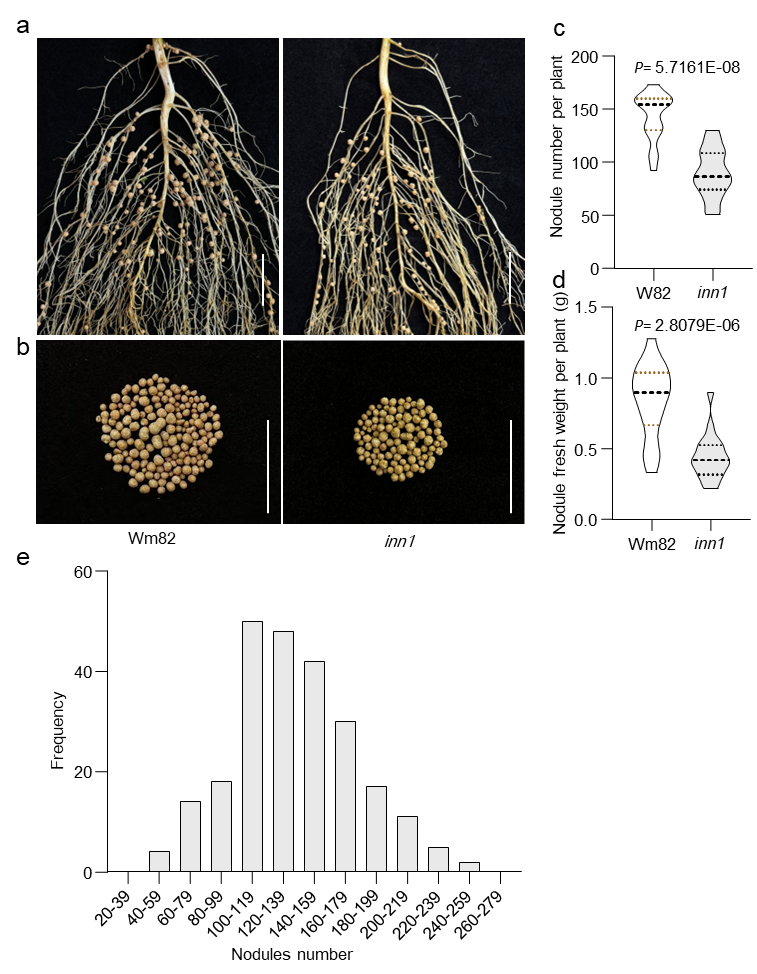


**Fig. S3. Nodule phenotypes of the F_2_ segregating population derived from the cross between Wm82 and *inn1*.**

(a, b) Root (a) and nodule (b) phenotypes of wild-type c.v. Wm82 and *inn1* at 21 dai. Scale bar = 2 cm. (c) Nodule number per plant for wild-type c.v. Wm82 and *inn1* (n = 18 plants). (d) Nodule fresh weight per plant for wild-type c.v. Wm82 and *inn1* (n = 18 plants). (e) Distribution of root-nodule number in individual plants from the F_2_ segregating population derived from the cross between Wm82 and *inn1*. For c and d, data are means ± SD. Two-tailed Student’s t-tests (*P* < 0.05) were used to compare means.


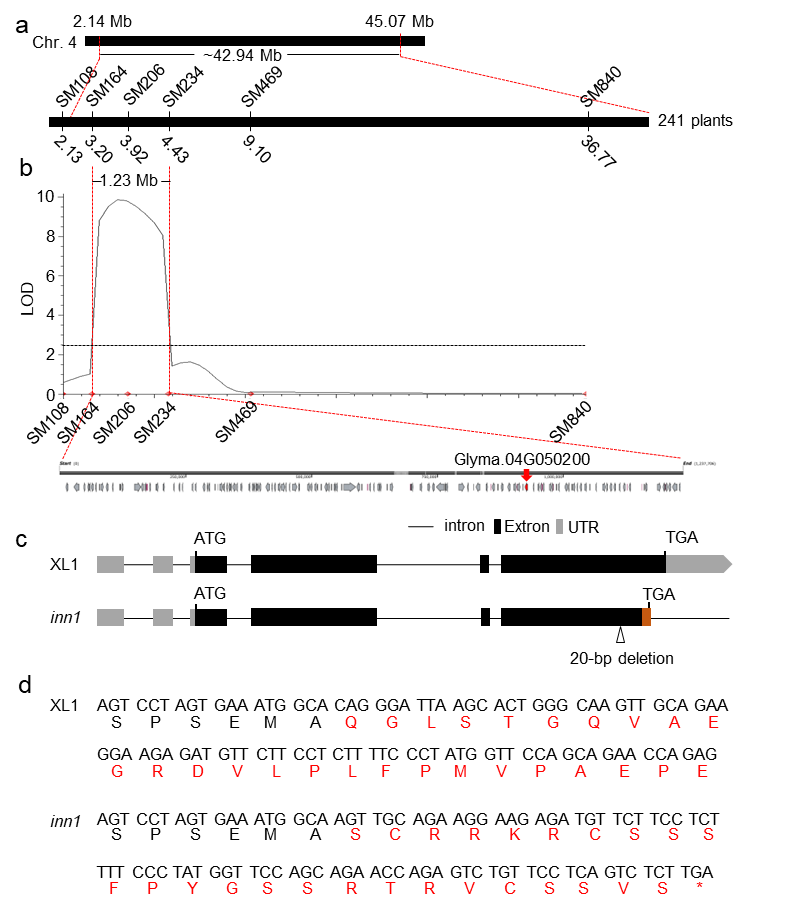


**Fig. S4. Genetic mapping of the *INN1* gene.**

(a) Zoom-in of a segment of chromosome 4 as depicted in panel A. Six polymorphic SSR markers were selected from the chromosome-4 interval identified by BSA-seq for genotyping in F_2_ individuals. The number below the tag name represents its physical location on the chromosome (in Mb). (b) Identification of *INN1* candidate genes through QTL mapping. The QTL had a maximum LOD of 9.84, which explained 17.35% of the variation. The candidate region on chromosome 4 was narrowed using IciMapping. The dashed line represents the threshold value of 2.5. The gene indicated by the red arrow represents the target gene. (c) Genomic structure of wild-type *INN1* and *inn1*. In the *inn1* mutant, a 20-bp deletion from the fourth exon results in a premature stop codon, which encodes a truncated protein 668 amino-acids long versus 715 for wild-type INN1. The brown squares represent nucleic acid sequences that differ from Wm82. (d) Predicted amino acid sequence changes in *INN1* and *inn1* from the indicated mutants and Wm82. Red letters indicate the nucleotide sequence differences between XL1 and *inn1*; asterisk, termination of translation.


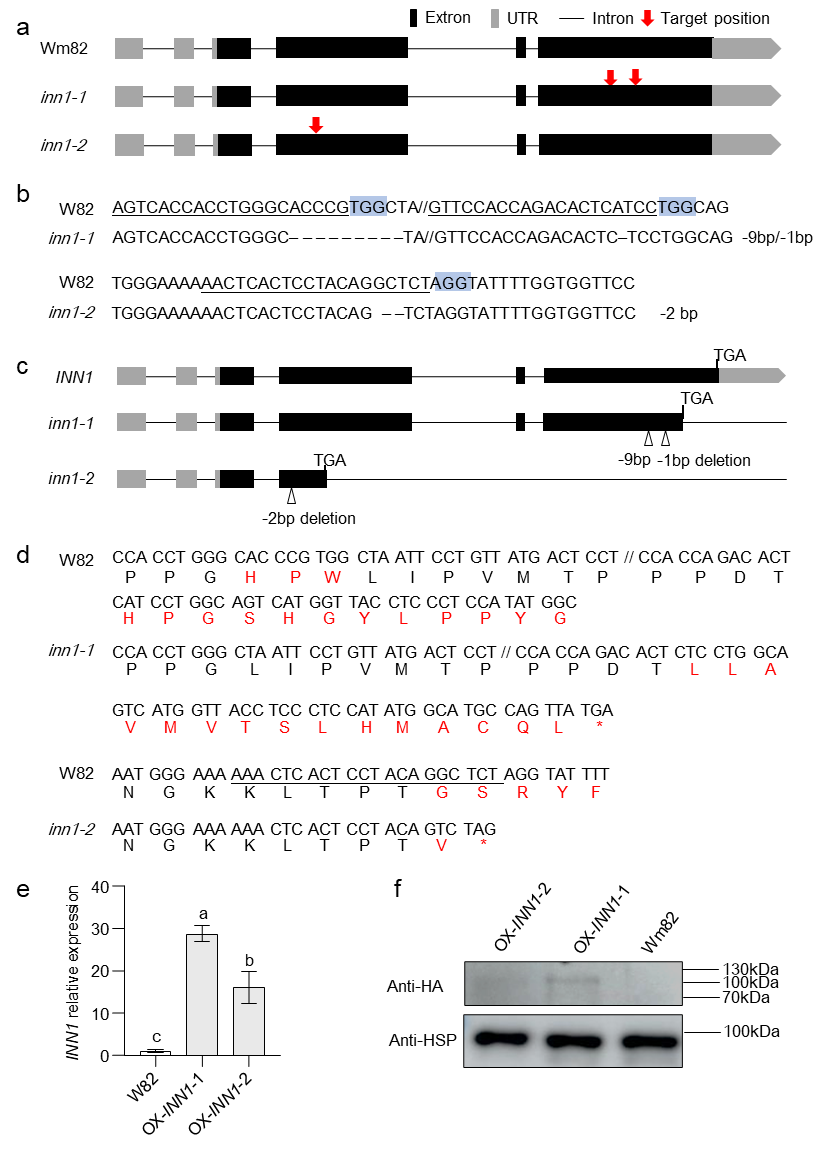


**Fig. S5. Generation and characterization of *INN1* mutants and transgenic over-expression lines.**

(a) *INN1* genomic structure depicting target sites in the second and fourth exons for CRISPR–Cas9 mutagenesis, resulting in *inn1-1* and *inn1-2* mutant alleles. (b) Genotypes of *inn1-1* and *inn1-2* mutant alleles. Sequence alignments of the sgRNA target sites in soybean cultivar ‘Williams 82’ (Wm82) and mutant alleles of *inn1-1* and *inn1-2*. The blue highlight indicates the protospacer-adjacent motif (PAM) NGG; underlined nucleotides represent the sgRNA target site. Dashes indicate mutated nucleotides. ‘//’ indicates the omission of an identical sequence between two target sites. (c) Genomic-structure changes in *inn1-1* and *inn1-2* alleles versus wild-type c.v. Wm82. (d) Predicted amino acid sequence changes in *inn1-1* and *inn1-2* from the indicated mutants and Wm82. Red letters indicate the amino acid differences between Wm82 and CRISPR–Cas9 mutagenesis line; asterisk, termination of translation. (e) RT–qPCR of *INN1* expression in 3-d old root samples isolated from wild-type c.v. Wm82 and two genetically independent transgenic *INN1–6xHA* over-expression lines. Data are the mean ± SE of n = 3 biological replicates and relative expression was normalized to *ACTIN11*. One-way ANOVA was used to determine statistical significance (P < 0.05) and different letters indicate statistically significant differences. (f) Western blot with anti-HA antibodies probing INN1–6xHA abundance in transgenic over-expression lines. 0.1 micrograms of total protein isolated from root was loaded onto a 8% gel and HSP was used as a loading control.


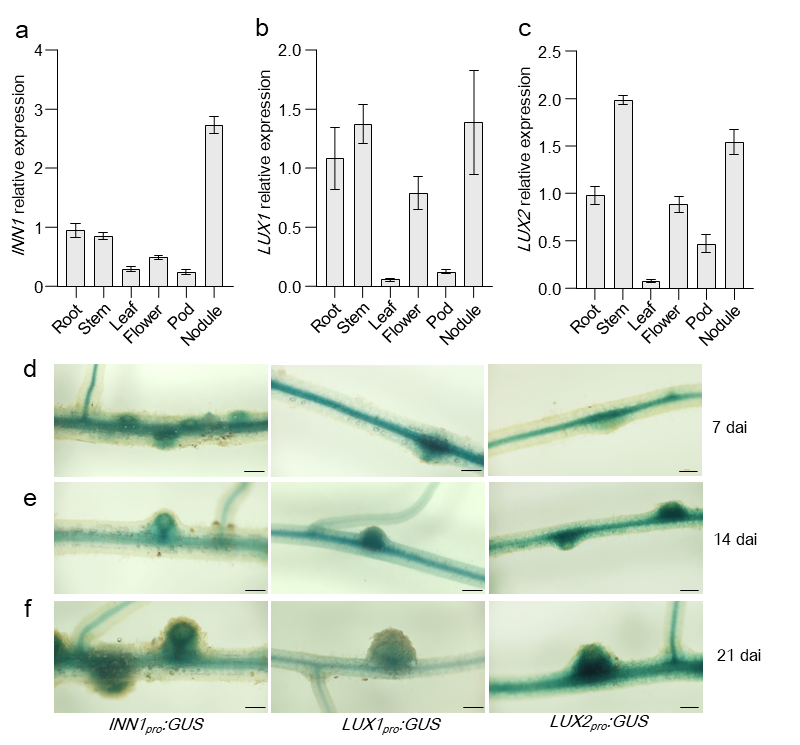


**Fig. S6. *INN1*, *LUX1* and *LUX2* are all expressed in several soybean tissues.**

(a–c) RT–qPCR of *INN1*, *LUX1* and *LUX2* expression patterns in different tissues harvested from 21-d old plants at ZT 12. Data are the mean ± SE of n = 3 biological replicates and relative expression was normalized to *ACTIN11.* (d–f) Histological GUS staining of transiently transgenic hairy roots expressing *INN1_pro_:GUS*, *LUX1_pro_:GUS* and *LUX2_pro_:GUS* reporters in the Wm82 background, observed in nodules at 7-d-old (d), 14-d-old (e), and 21-d-old (f) after inoculation. Scale bar = 5 μm.


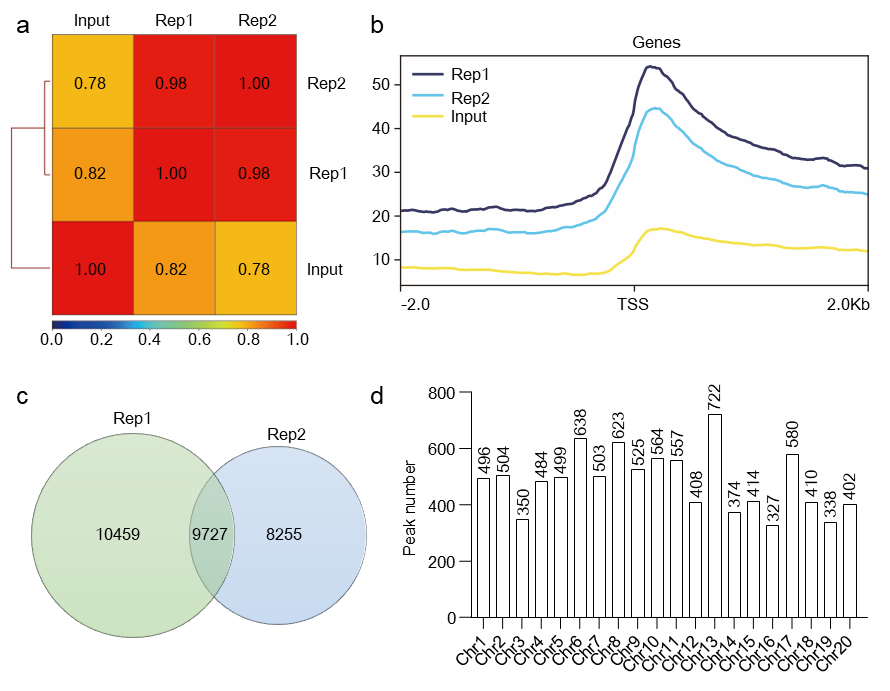


**Fig. S7. Identification of LUX Binding Sites via ChIP-seq Data Analysis.**

(a) Correlation between the input sample and replicates 1 and 2 (rep1 and rep2). (b) ChIP-seq signal intensity surrounding the transcription start sites (TSS) of enriched genes within a ±2 kb window. (c) Number of overlapping peaks identified between the two IP samples in the LUX2 ChIP-seq analysis. (d) Distribution of 9,727 peaks across the 20 chromosomes, showing only the peaks located on the 20 chromosomes.


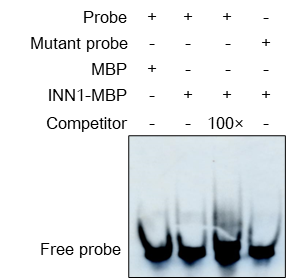


**Fig. S8. INN1–MBP does not directly bind to the *ENOD40* promoter in the EMSA.**


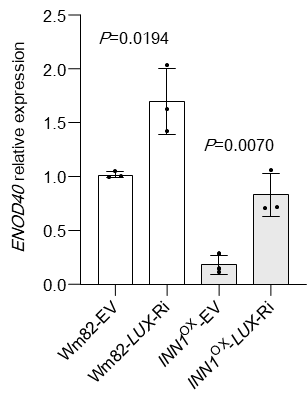


**Fig. S9. *ENOD40* expression levels in different transgenic hairy roots lines.**

RT–qPCR of *ENOD40*expression levels in transgenic hairy roots carrying the empty vector (EV) and *LUX* RNAi vector (n = 3 biological replicates ± S.E. with individual plant values shown as dots). Relative expression was normalized to *ACTIN11*. *P* < 0.05, two-tailed Student’s t-test.


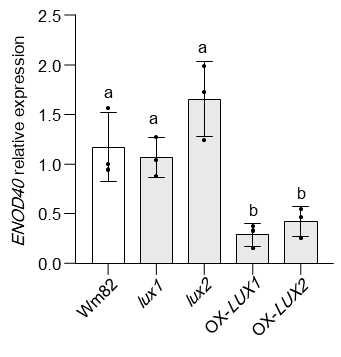


**Fig. S10. *ENOD40* expression levels in different soybean genotypes.**

*ENOD40* expression in 3-d old root isolated from wild-type c.v. Wm82, *lux1*, *lux2,* and transgenic *LUX1–FLAG* and *LUX2–FLAG* lines following rhizobial inoculation. Different letters represent statistically significant differences determined by one-way ANOVA, *P* < 0.05. Data are the mean ± SD of n = 3 biological replicates and relative expression was normalized to *ACTIN11.*


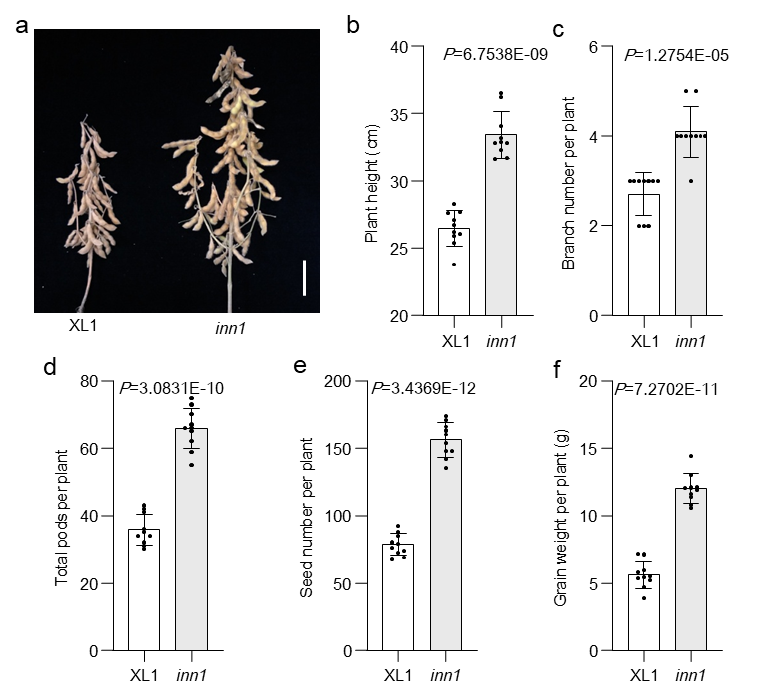


**Fig. S11. Characteristics of mature plants of XL1 and *inn1* mutant.**

(a) Plant phenotypes of XL1 and *inn1* mutants at maturity, Scale bar, 5 cm. (b–f) plant height (b), branch number (c), total pods per plant(d), seed number per plant(e), and grain weight per plant (f) of XL1 and *inn1* mutants. Plants were grown in Guangzhou (23°S, 112°E) under natural SD conditions. For b to f, data are means ± SD with individual plant values shown as dots (n = 10). P < 0.05, two-tailed Student’s t-test.


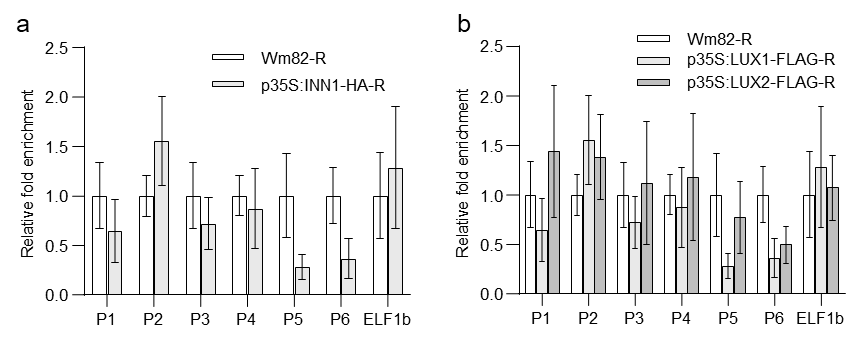


**Fig. S12. The INN1–LUX complex does not bind to the *ENOD40* promoter in the absence of rhizobial inoculation**

(a) ChIP–qPCR of INN1–HA binding to the *ENOD40* promoter in stable-transgenic lines under non-inoculated conditions. (b) LUX1–FLAG and LUX2–FLAG binding to the *ENOD40* promoter in stable-transgenic lines under non-inoculated conditions. The *ELF1b* promoter was used as a negative control.
